# Gen-miR-5 derived from *Gentianella acuta* inhibits PFKP to prevent fibroblast activation and alleviate myocardial fibrosis

**DOI:** 10.3389/fphar.2025.1578877

**Published:** 2025-05-02

**Authors:** Hongyao Ge, Zhenyu Du, Weizhe Liu, Letian Wang, Junyang Li, Gaoshan Yang, Aiying Li

**Affiliations:** ^1^ Institute of Chinese Materia Medica, Shanghai University of Traditional Chinese Medicine, Shanghai, China; ^2^ Hebei Key Laboratory of Chinese Medicine Research on Cardio-cerebrovascular Disease, Shijiazhuang, Hebei, China; ^3^ Hebei Higher Education Institute Applied Technology Research Center on TCM Development and Industrialization, Shijiazhuang, Hebei, China; ^4^ Department of Biochemistry and Molecular Biology, College of Pharmacy, Hebei University of Chinese Medicine, Shijiazhuang, Hebei, China; ^5^ Department of Cardiology, The Fifth People’s Hospital of Shanghai, Fudan University, Shanghai, China

**Keywords:** plant-derived miRNAs, PFKP, lactate, fibroblast activation, myocardial fibrosis

## Abstract

**Introduction:**

Myocardial fibrosis (MF) is a key pathological change in heart failure, and lactate a product of glycolysis, is an important component affecting the process of MF. miRNAs derived from *Gentianella acuta* (*G. acuta*) have been shown to effectively treat cardiac remodeling. However, whether *G. acuta*-derived Gen-miR-5 can effectively improve MF remains to be elucidated. This study seeks to explore the pharmacological effects and underlying molecular mechanisms of Gen-miR-5 in the context of Angiotensin II (Ang II) -induced MF.

**Methods:**

A mouse model of MF was established by subcutaneous infusion of Ang II using osmotic pumps, and then administration of Gen-miR-5 by injection. The effects of Gen-miR-5 in reducing MF and exerting cardioprotective actions were evaluated through pathological morphological analysis and echocardiography. The targeting effect of Gen-miR-5 on PFKP was assessed through dual-luciferase reporter gene assays. Cardiac fibroblasts (CFs) migration abilities were evaluated through wound healing assay and transwell assays. Additionally, the role of Gen-miR-5 in fibroblast activation was investigated using gain- and loss-of-function experiments, and immunofluorescence.

**Results:**

This study identified six novel specific miRNAs in *G. acuta*, among which Gen-miR-5 can be absorbed by mice, stably exists in cardiac tissue, and targets the PFKP 3’ UTR to exert cross-kingdom regulatory effects. PFKP, as a key rate-limiting enzyme in the glycolytic pathway, increases lactate accumulation and promotes the proliferation and migration of CFs, thereby facilitating the development of MF. In contrast, Gen-miR-5 alleviates MF by inhibiting this process.

**Discussion:**

In conclusion, we have elucidated for the first time the pharmacological effects of Gen-miR-5, derived from *G. acuta*, in inhibiting MF. Gen-miR-5 exerts its cardioprotective effects by targeting and inhibiting the expression of the key glycolytic enzyme PFKP, induced by Ang II, regulating lactate metabolism in fibroblast, and preventing the transformation of fibroblasts into myofibroblasts, ultimately alleviating MF. This study demonstrates that Gen-miR-5 is a potential therapeutic agent for improving cardiac remodeling.

## Graphical Abstract

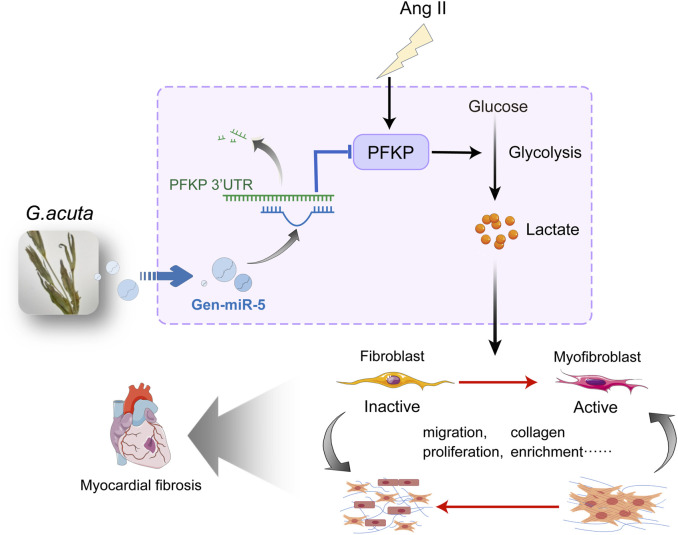

## Introduction

MF commonly occurs in cardiovascular diseases (Gourdie et al., 2016). Pressure overload to the heart induce structural and functional changes, leading to cardiac remodeling (Frantz et al., 2022), with MF serving as a significant hallmark of this process (Peng et al., 2024). A series of biological behavior changes such as abnormal proliferation and migration of CFs are closely related to stress overload (Liu et al., 2023; Souders et al., 2009; Tallquist and Molkentin, 2017). Initially, fibroblasts undergo phenotypic transformation under stress, initiating “reparative” fibrosis by proliferating to replace dead cardiomyocytes (Weber et al., 2013). Uncontrolled chronic activation, such as pressure overload, triggers the production and accumulation of collagen in the extracellular matrix and stroma (Tan et al., 2020), leading to cardiac dysfunction and adverse effects on the body (Humeres et al., 2024; Khalil et al., 2017). Therefore, focusing on inhibiting fibroblast proliferation and migration may represent a key strategy for treating cardiovascular diseases.

Under pathological conditions, the cardiac metabolic pathway shifts from fatty acid oxidation to glycolysis (Keceli et al., 2022; Ranjbarvaziri et al., 2021). Glycolysis promotes MF by activating CFs (Hailiwu et al., 2023), further inducing cardiac remodeling (Ma et al., 2024). PFKP as a key enzyme in glycolysis, plays a vital role in metabolic remodeling by enhancing the levels of glycolytic products, such as pyruvate and lactate, which aggravate cardiac contractile dysfunction (Bertero and Maack, 2018), positioning PFKP as a novel player in cardiac remodeling (Vigil Garcia et al., 2021). Lactate is one of the key products of glycolysis and plays a critical role in the progression of various diseases (Yang et al., 2022; Yang et al., 2022; Yang et al., 2020). During pressure overload in the heart, lactate levels increase significantly (Cluntun et al., 2021; Dai et al., 2020). Recent studies have confirmed that lactate promotes MF during myocardial infarction by enhancing EndoMT, thereby exacerbating cardiac dysfunction (Fan et al., 2023). Both PFKP and lactate are critical contributors to cardiovascular diseases. However, their potential connection in the pathogenesis of MF remains unclear.

Plant-derived miRNAs exhibit enhanced stability due to the antioxidative 2′-O-methylation modifications on their terminal nucleotides and can be absorbed by mammals through the SIDT1 protein in gastric mucosal epithelial cells (Chen et al., 2021). An increasing number of studies have demonstrated that miRNAs derived from traditional Chinese medicine (TCM) can cross-kingdom regulate gene expression, contributing to therapeutic effects and providing new evidence and insights into TCM-based disease treatments (Chin et al., 2016; Ji et al., 2019; Yang et al., 2020; Zhao et al., 2023). *Gentianella acuta*, also known as bitter gentian, belongs to the Gentianaceae family and the genus Gentianella. According to the Compendium of Materia Medica, it is described as “bitter, astringent, cold, and non-toxic.” This species is recorded in Flora of China and The Mongolian Pharmacopoeia, where it is used in Mongolian and Tibetan medicine. It is commonly referred to as “Agu-te-Qiqige” in Mongolian and “Sangdige” in Tibetan. This herb is known for its therapeutic properties, including clearing heat, promoting bile excretion, and treating jaundice, and is frequently used to treat angina. Additionally, some reports suggest that long-term chewing or drinking it as tea may help treat and prevent cardiovascular diseases (Ding et al., 2017; Sun et al., 2021; Zhou et al., 2022). *Gentianella acuta* is known to contain active components such as iridoids and xanthones, but the cardioprotective effects of these compounds cannot be fully explained by any single component. In contrast, *G. acuta*-derived miRNAs, as novel bioactive molecules, exhibit higher targeting specificity and regulatory precision, enabling more precise therapeutic effects through gene expression modulation. Our previous studies have revealed that *G. acuta* exhibits significant cardiovascular protective effects (Yang et al., 2020; Zhao et al., 2024; Zhou et al., 2023), and its derived Gen-miR-1 effectively inhibits the development of CF by alleviating cardiac inflammation (Zhang et al., 2023). Additionally, we found that Gen-miR-5 derived from *G. acuta* alleviates cardiac hypertrophy by targeting PFKP; however, its underlying mechanisms in inhibiting MF remain unclear. However, there have been no reported studies demonstrating that *G. acuta*-derived miRNAs can suppress proliferation and migration by regulating lactate metabolism in cardiac fibroblasts. Therefore, further investigation into the regulatory effects of Gen-miR-5 on fibroblast proliferation and activation is of great significance for confirming the cardioprotective effects of *G. acuta*.

In this study, we report that Ang II promotes the proliferation and migration of CFs and their transformation into activated myofibroblasts by activating PFKP, which synergizes with lactate to facilitate MF. Meanwhile, Gen-miR-5 derived from *G. acuta* mitigates excessive fibroblast proliferation and migration and reduces MF by targeting inhibition of PFKP expression.

## Materials and methods

### Reagents

Gen-miR-5 and RNA Fluorescence *in Situ* Hybridization (FISH) assay kit were purchased from GenePharma (Suzhou, China). Ang II was purchased from Glpbio (Montclair, USA). Dual Fluorokinase Reporter Gene Assay System was purchased from Promega (Wisconsin, USA). Antibodies against PFKP, Ki67, MMP9, MMP2, Cyclin D1, PCNA, and GAPDH were purchased from Proteintech (Wuhan, China). Antibodies against Collagen III, Collagen I, and α-SMA were purchased from Cell Signaling (Massachusetts, USA).

### Animal model preparation

In this study, male C57BL/6 mice, 6–8 weeks old and weighing between 19 and 23 g, were utilized (Approval No. DWLL202203114, 21 March 2022). Throughout the experiment, all animals were allowed unrestricted access to food and water. The mice were randomly divided into four groups, with six mice assigned to each group. Ang II was delivered *via* subcutaneously implanted osmotic pumps (RWD, China) at a dose of 1.5 mg/kg/day for 14 consecutive days, while the control group received an equal volume of saline. Gen-miR-5 was administered orally at 2-day intervals during the experimental period.

### Echocardiography

Echocardiography was performed on day 14 using the Vevo 2100 high-resolution small animal ultrasound imaging system (Visual Sonics, Toronto, Ontario, Canada). After anesthetizing the mice with a mixture of oxygen and isoflurane (3%), M-mode was used to measure the left ventricular posterior wall thickness at end-systole and end-diastole (LVPW), ejection fraction (EF), and fractional shortening (FS) to assess cardiac structure and function. At least three independent cardiac cycles were analyzed, and the average value was calculated.

### High-throughput sequencing and bioinformatics methods

A total of 3 μg of RNA was isolated from *G. acuta* and utilized as the input material for the plant miRNA database. New miRNAs were predicted based on the hairpin structures of miRNA precursors. Custom scripts were used to obtain the initial loci of a certain length, count all identified miRNAs, and analyze base bias at each locus. High-throughput sequencing and bioinformatics of *G. acuta* were independently repeated and validated by qRT-PCR.

### Sodium periodate oxidation of RNA

Total RNA was extracted from the heart and plasma using the Trizol method. After measuring the concentration, 20 μg of RNA was added to 95 μL of 10 mM NaIO_4_ and incubated in the dark at 0°C for 40 min. Subsequently, 1 mL of anhydrous ethanol and 1 μL of glycogen were added to the oxidized RNA, followed by an ice bath for 20 min and centrifugation. The supernatant was discarded, and the pellet was washed sequentially with anhydrous ethanol and 75% ethanol. Finally, the pellet was dissolved in DEPC-treated water for qRT-PCR analysis.

### Real-time quantitative PCR analysis

Total RNA from HCFs and cardiac tissue, as well as RNA from plasma, were extracted using the Total RNA Kit II (Omega, China) and the miRNeasy Serum/Plasma Kit. After measuring the concentration using a spectrophotometer, 20 μg of RNA was added to 95 μL of NAIO_4_ (10 mM) and incubated for 40 min at 0°C in an environment protected from light. To the oxidized RNA, 1 mL of anhydrous ethanol and 1 μL of glycogen were added, and high-speed centrifugation was performed after 20 min in an ice bath. The supernatant was aspirated and then precipitated and washed by adding anhydrous ethanol and 75% ethanol, respectively. Finally, DEPC water was added to dissolve the precipitate. qRT-PCR was performed using the miScript PCR system (Qiagen,218,161).

### Western blot analysis

Heart tissues and HCFs were lysed in RIPA, PMSF, cocktail, and phosphatase inhibitors. After extraction, samples were boiled in 5×loading buffer for denaturation. Proteins were separated through SDS-PAGE and transferred onto PVDF membranes (Millipore). Incubate with the corresponding primary antibody at 4°C overnight, including anti-PFKP, anti-Collagen III, anti-Collagen I, anti-α-SMA, anti-MMP9, anti-MMP2, anti-Cyclin D1, anti-PCNA, and anti-GAPDH. After washing with TBST and TBS, the membranes were treated with HRP-conjugated secondary antibodies (HRP-labeled goat anti-rabbit IgG (H + L) and HRP-labeled goat anti-mouse IgG (H + L), both from ZSGB-BIO) for 1.5 h at room temperature. Protein bands were visualized using a chemiluminescent substrate (Abbkine, China) and detected with a chemiluminescence imaging system.

### Histological analysis

Freshly isolated cardiac tissues were fixed with 4% paraformaldehyde and then 5-μm-thick tissue sections were made. Heart sections were stained with hematoxylin and eosin, Masson’s trichrome stain, and Sirius Red stain. Panoramic scanning imaging of HE-stained sections was performed by panoramic scanning microscopy to observe the morphological changes of cardiac tissues. The stained completed Masson sections were observed using a Leica microscope to compare the deposition of collagen fibers between groups.

### Immunohistochemistry

The prepared paraffin sections of heart tissue were baked at 65°C for 1 h to melt the paraffin. Sections were quickly placed in xylene for dewaxing and then rehydrated in different concentration gradients of ethanol (100%, 90%, 80%, 70%). After washing with PBS, the sections were placed in 0.01 mol/L sodium citrate at 100°C for antigen repair. Sections restored to room temperature were washed with PBS and then 3% H_2_O_2_ was added dropwise to reduce the effect of endogenous peroxidase, and the incubation was completed before being closed with 10% goat serum. Finally, diluted primary antibody (MMP2, Proteintech; PCNA, Proteintech) was added dropwise and incubated at 4°C overnight. On the following day, the sections were returned to room temperature before being washed with PBS, and after the washing completed the secondary antibody was added dropwise and incubated at room temperature. Then the HRP-labeled streptomycin working solution was added dropwise for incubation, washed with PBS, and then added dropwise with DAB colorant for color development, at this time, attention should be paid to the degree of color development to avoid overstaining. Then drops of hematoxylin were added to re-stain the nuclei. After staining is complete the sections are placed in 1% hydrochloric acid alcohol for differentiation and bluing, which takes only a few seconds. The slices were then placed in different concentration gradients of ethanol (70%, 80%, 90%, 100%) and xylene for dehydration and transparency, respectively. A final drop of neutral gum was added to seal the film. Imaging photographs were taken using a Leica microscope photosystem.

### Immunofluorescence

The prepared paraffin sections of heart tissue were baked at 65°C to melt the paraffin. The sections were then sequentially placed in xylene and different concentrations of ethanol (100%, 90%, 80%, 70%) for dewaxing and rehydration. Sections were washed with 0.5% Triton X-100 and then closed with 10% goat serum dropwise for 30 min. At the end of the blocking, diluted primary antibodies (Ki67, Proteintech) were added dropwise and incubated overnight in a 4°C environment. On the following day, the sections were washed with 0.5% Triton X-100 and PBS after being returned to room temperature and then incubated dropwise with fluorescent secondary antibody for 1 h at room temperature. The nuclei were then stained with drops of DAPI after washing with 0.5% Triton X-100 and PBS, respectively. After staining was completed and washed with PBS, the slices were sealed by dropwise addition of anti-fluorescence quenching sealer. Finally, an anti-fluorescence quenching sealer was added dropwise.

HCF cells were inoculated on sterile cell crawls and the cells were treated. Cells were washed with PBS and then fixed with 4% paraformaldehyde. Cells after completion of fixation were permeabilized with 0.2% Triton X-100. After washing with PBS, 5% goat serum was added dropwise for containment. Dropwise addition of diluted primary antibody (Ki67, Proteintech; Collagen III, Servicebio) was incubated at 4°C overnight. On the following day, cells were washed with PBS and then fluorescent secondary antibody was added dropwise and incubated for 1.5 h at room temperature. Then DAPI was added dropwise to stain the cell nuclei. Finally, the film was sealed by the dropwise addition of anti-fluorescence quenching sealer.

### Dual-luciferase reporter gene assay

The dual-luciferase reporter assay system (Promega, USA) was employed to confirm the regulatory interaction between PFKP and Gen-miR-5. HCFs were transfected with PFKP wild-type or mutant plasmids using Lipofectamine 3000. After 24 h of transfection, luciferase activity was assessed across the groups using the dual-luciferase reporter assay system.

### RNA fluorescence *in situ* Hybridization (FISH)

Paraffin-embedded sections were heated to melt the paraffin. The sections were then deparaffinized by sequential immersion in xylene and a graded ethanol series. After washing twice with PBS, 37°C preheated Proteinase K working solution was added for incubation. Blocking solution was applied for 30 min, followed by a rinse with 2 × Buffer C. Preheated denaturation solution at 78°C was then added. The heart tissue was dehydrated and dried using a graded ethanol series. The prepared probe mixture was applied and incubated for 12–16 h. The next day, the sections were washed with washing solution and then rinsed with 2 × Buffer C. After washing, DAPI staining solution was applied. Finally, the sections were mounted with an anti-fade mounting medium.

### Wound healing assay

Fibroblasts were cultured to 70%–80% confluence, and a sterile pipette tip was used to create a straight scratch across the cell monolayer. After washing with PBS, a fresh culture medium was added, followed by treatment with Ang II and Gen-miR-5. Images of the scratch area were captured at 0 and 24 h post-scratching to evaluate fibroblast migration into the wound area. The migration capacity of the cells was quantified by calculating the area of the scratch region.

### Determination of lactic acid content

Lactic acid content in cardiac tissue was determined using the D-Lactic Acid/Lactate (LA) Colorimetric Assay Kit (Elabscience) according to the manufacturer’s instructions. Protein concentration was determined after homogenizing the heart tissue with PBS. Add standards, protein samples to be tested, enzyme working solution, and other reagents to the 96-well plate sequentially. After addition, measurements were taken using a microplate reader at a wavelength of 530 nm. Finally, the data were calculated and counted based on the standard curve and OD values of each well.

### Statistical analysis

Data analysis was conducted using IBM SPSS 23.0 (SPSS Inc., Chicago, USA) and GraphPad Prism 8 (GraphPad, San Diego, USA). Results were expressed as mean ± standard deviation (SEM). A P-value of less than 0.05 was considered statistically significant.

## Results

### Gen-miR-5 derived from *Gentianella acuta* can enter the mouse body following its exogenous administration

**FIGURE 1:**
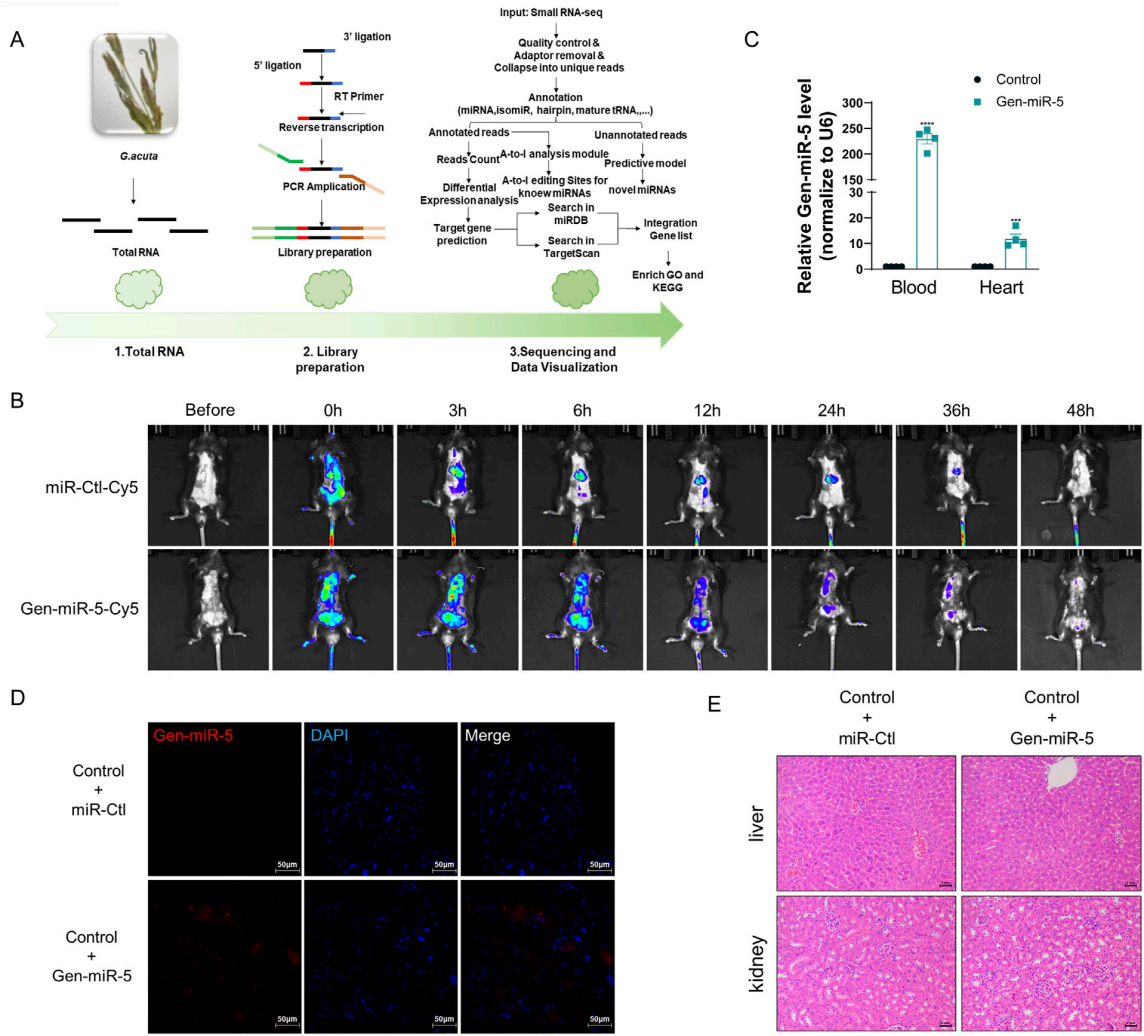
Gen-miR-5 specifically present in *G.acuta* might enter the mouse body after exogenous administration to mice **(A)** Flowchart of High-Throughput Sequencing and Bioinformatics Analysis. **(B)** Schematic illustration of *in vivo* imaging of Gen-miR-5 in mice. **(C)** miRNA was extracted from blood and cardiac tissues and treated with sodium periodate, followed by qRT-PCR to assess the expression levels of Gen-miR-5. Values are expressed as mean ± SEM. ^***^ *P* < 0.001, ^****^ *P* < 0.0001 compared to the corresponding control group (n = 4). **(D)** Gen-miR-5 in cardiac tissue was detected using RNA fluorescence *in situ* hybridization. Red fluorescence indicates Gen-miR-5 and blue fluorescence marks the nuclei. Scale bar = 50 μm. **(E)** H&E staining of mouse liver and kidney tissue sections. Scale bar = 2 mm.

*Gentianella acuta* is increasingly recognized for its significant cardioprotective effects. To discover more efficient and targeted active components for cardiovascular diseases within *G. acuta*, we used high-throughput sequencing to identify and screen its miRNAs (Figure 1A). Six of these miRNAs have not been found in any other plants, suggesting they might be key to the cardioprotective action of *G. acuta*. Among these, Gen-miR-5, which is highly abundant, has caught our attention. We first assessed the absorption and metabolic process of Gen-miR-5 in mice. First, Gen-miR-5 agomir was synthesized *in vitro* and fluorescently labeled. The results of imaging small animals *in vivo* showed that Gen-miR-5 was widely expressed in mice after exogenous administration. Over time, Gen-miR-5 gradually enriched into the heart, reaching the maximum expression abundance at 6 h, and then gradually decreased until Gen-miR-5 was completely metabolically degraded at 48 h (Figure 1B). Additionally, considering the antioxidative 2′-O-methylation characteristic of the 3′ ends of plant-derived miRNAs, we treated mouse blood and cardiac tissues with sodium periodate and screened for Gen-miR-5 from *G. acuta* using qRT-PCR and the miScript PCR system. We found high expression levels of Gen-miR-5 in both the blood and heart tissues of mice (Figure 1C). Meanwhile, designing a specific probe according to the sequence of Gen-miR-5, we confirmed the presence of Gen-miR-5 in cardiac tissues through FISH (Figure 1D). To further explore whether Gen-miR-5 has any toxic side effects and adverse reactions. We performed histopathological examinations on mouse liver and kidney tissues. No pathological changes were observed in the liver or kidneys, indicating the reliability and safety of Gen-miR-5 for treating cardiovascular diseases (Figure 1E).

### Gen-miR-5 can significantly improve pressure overload-induced MF

**FIGURE 2:**
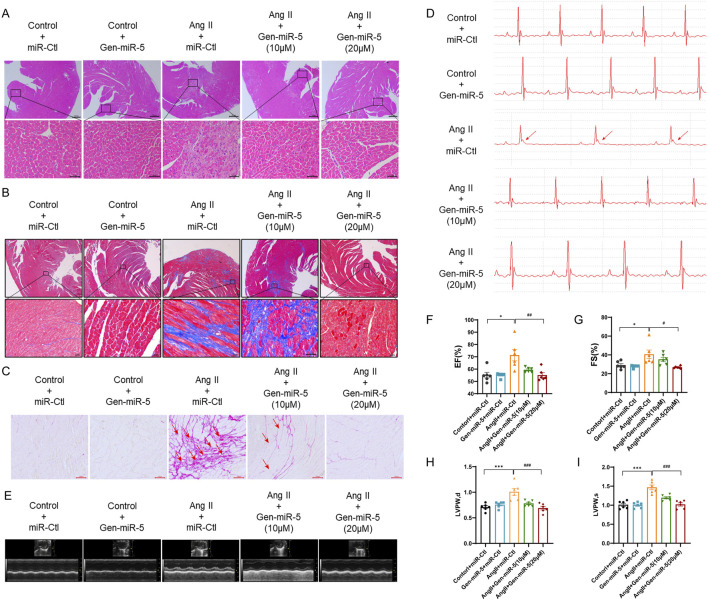
Gen-miR-5 inhibits myocardial fibrosis C57BL/6 mice were subcutaneously infused with Ang II (1.5 mg/kg/day) or an equivalent volume of saline, continuously for 2 weeks, while Gen-miR-5 was administered orally every 2 days **(A)** H&E staining of cross-sectional slices of cardiac tissue. The white arrow indicates the structural changes and inflammatory infiltration occurring in the cardiac tissue. Scale bar = 20 μm. **(B)** Masson’s trichrome staining. The white arrow indicates the deposition of collagen fibers. Scale bar = 20 μm. **(C)** Sirius Red staining. The red arrow indicates the deposition of collagen fibers. Scale bar = 100 μm. **(D)** The electrocardiogram represents the image. The red arrow indicates the ST-segment elevation. **(E)** Representative echocardiogram images. **(F–I)** Ejection fraction (EF), fractional shortening (FS), and left ventricular posterior wall (LVPW) thickness at end-systole and end-diastole. Values are expressed as mean ± SEM. ^*^ *P* < 0.05, ^***^ *P* < 0.001 compared to the corresponding control group. ^#^ *P* < 0.05, ^###^ *P* < 0.001 compared to the corresponding Ang II + miR-Ctl group (n = 6).

MF, a major manifestation of cardiac remodeling, has always been a focus of researchers. To explore the preventive and therapeutic effects of Gen-miR-5 on Ang II-induced MF, we implanted osmotic pumps in mice for continuous infusion of Ang II, establishing a model of pressure overload-induced MF. Low (10 μM) and high (20 μM) doses of Gen-miR-5 were administered to identify the optimal dose. HE staining revealed that Ang II caused disarray in the arrangement of cardiac cells and pathological changes such as inflammatory infiltration. However, treatment with Gen-miR-5 significantly restored the histopathological architecture of the cardiac tissues, with the high dose (20 μM) of Gen-miR-5 showing a more pronounced effect (Figure 2A). Additionally, Masson’s trichrome and Sirius Red staining revealed that Gen-miR-5 significantly reduces the extensive collagen fiber deposition in the cardiac tissues of mice treated with Ang II (Figures 2B,C). Moreover, electrocardiogram (ECG) results showed that after Ang II stimulation, the ST segment was significantly elevated, the QRS wave amplitude was reduced, and the heart rate was significantly reduced, indicating that the mice exhibited acute myocardial infarction. Furthermore, Gen-miR-5 significantly improved the pathological changes in the ECG induced by Ang II stimulation (Figure 2D). Furthermore, echocardiographic assessment of cardiac function showed that the ventricular cavity of the Ang II group mice was significantly smaller, and the cardiac structure was abnormally changed (Figure 2E), while the use of Gen-miR-5 decreases the Ang II-induced changes in ejection fraction (EF), fractional shortening (FS), and left ventricular posterior wall thickness (LVPW), thereby improving cardiac function and mitigating heart damage (Figures 2F-I). The above experimental results show that 20 μM Gen-miR-5 treatment resulted in better outcomes, and thus, this concentration was applied in subsequent experiments. These findings suggest that Gen-miR-5 effectively ameliorates stress-induced MF.

### Gen-miR-5 effectively inhibits CFs activation

**FIGURE 3:**
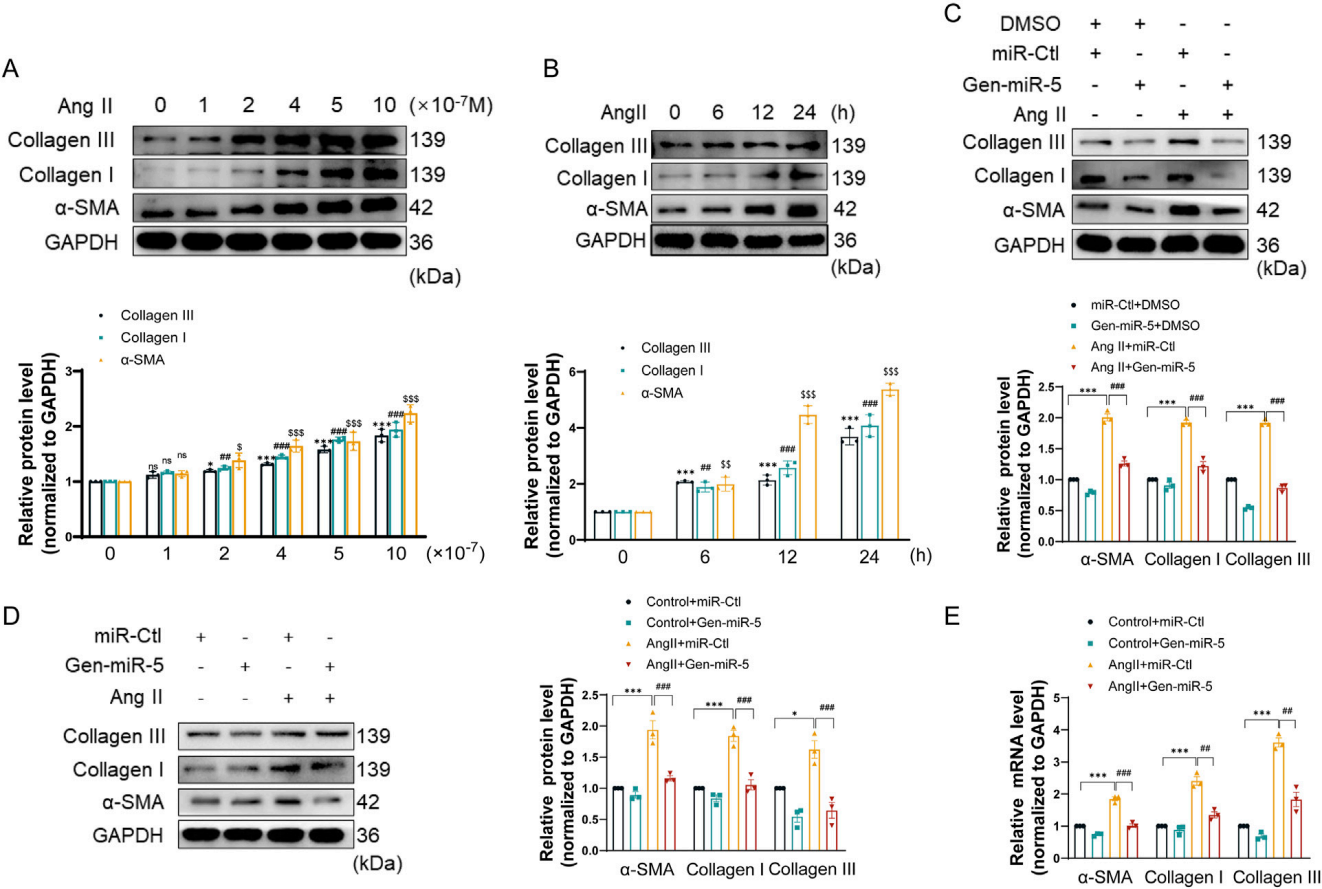
Gen-miR-5 inhibits the activation of cardiac fibroblasts **(A)** After treating cardiac fibroblasts with varying doses of Ang II for 24 h, protein expression levels of Collagen III, Collagen I, and α-SMA were assessed using Western blot (n = 3). **(B)** Cardiac fibroblasts were treated with Ang II (1 μM) at specified times, and the protein expression levels of Collagen III, Collagen I, and α-SMA were assessed using Western blot (n = 3). **(C)** Protein expression levels of Collagen III, Collagen I, and α-SMA in cardiac fibroblasts were assessed using Western blot (n = 3). **(D)** Protein expression levels of Collagen III, Collagen I, and α-SMA in cardiac tissue were measured using Western blot (n = 3). **(E)** mRNA levels of Collagen III, Collagen I, and α-SMA in cardiac tissue were measured using qRT-PCR (n = 3). Values are expressed as mean ± SEM. ^*^ *P* < 0.05, ^***^ *P* < 0.001, ^##^ *P* < 0.01, ^###^ *P* < 0.001, ^$^ *P* < 0.05, ^$$^ *P* < 0.01, ^$$$^ *P* < 0.001 compared to the corresponding control group.

MF is primarily characterized by the excessive accumulation and irregular distribution of collagen fibers resulting from the activation of CFs. To evaluate the impact of Ang II on CF activation, CFs were exposed to different concentrations of Ang II. The results showed that Ang II upregulates the expression of Collagen III, Collagen I, and α-SMA in a dose-dependent manner in CFs (Figure 3A). Additionally, treating CFs with Ang II at different times demonstrated that Ang II upregulates the expression of myofibroblast activation protein in a time-dependent manner (Figure 3B). The above results suggest that Ang II successful induction of CFs activation. Next, we transfected CFs with Gen-miR-5 and treated them with Ang II to investigate the effects of Gen-miR-5 on CFs activation. The results showed that Gen-miR-5 significantly inhibited the Ang II-induced expression of Collagen III, Collagen I, and α-SMA (Figure 3C). At the same time, we also assessed the expression of MF markers in cardiac tissues. The results indicated that Gen-miR-5 significantly reduced the expression of Collagen III, Collagen I, and α-SMA induced by Ang II (Figure 3D). qRT-PCR analysis confirmed that the mRNA levels of Collagen III, Collagen I, and α-SMA were consistent with changes in protein expression (Figure 3E). These findings demonstrate that Gen-miR-5 significantly inhibits Ang II-induced CFs activation.

### Gen-miR-5 inhibits Ang II-induced proliferation and migration of CFs

**FIGURE 4:**
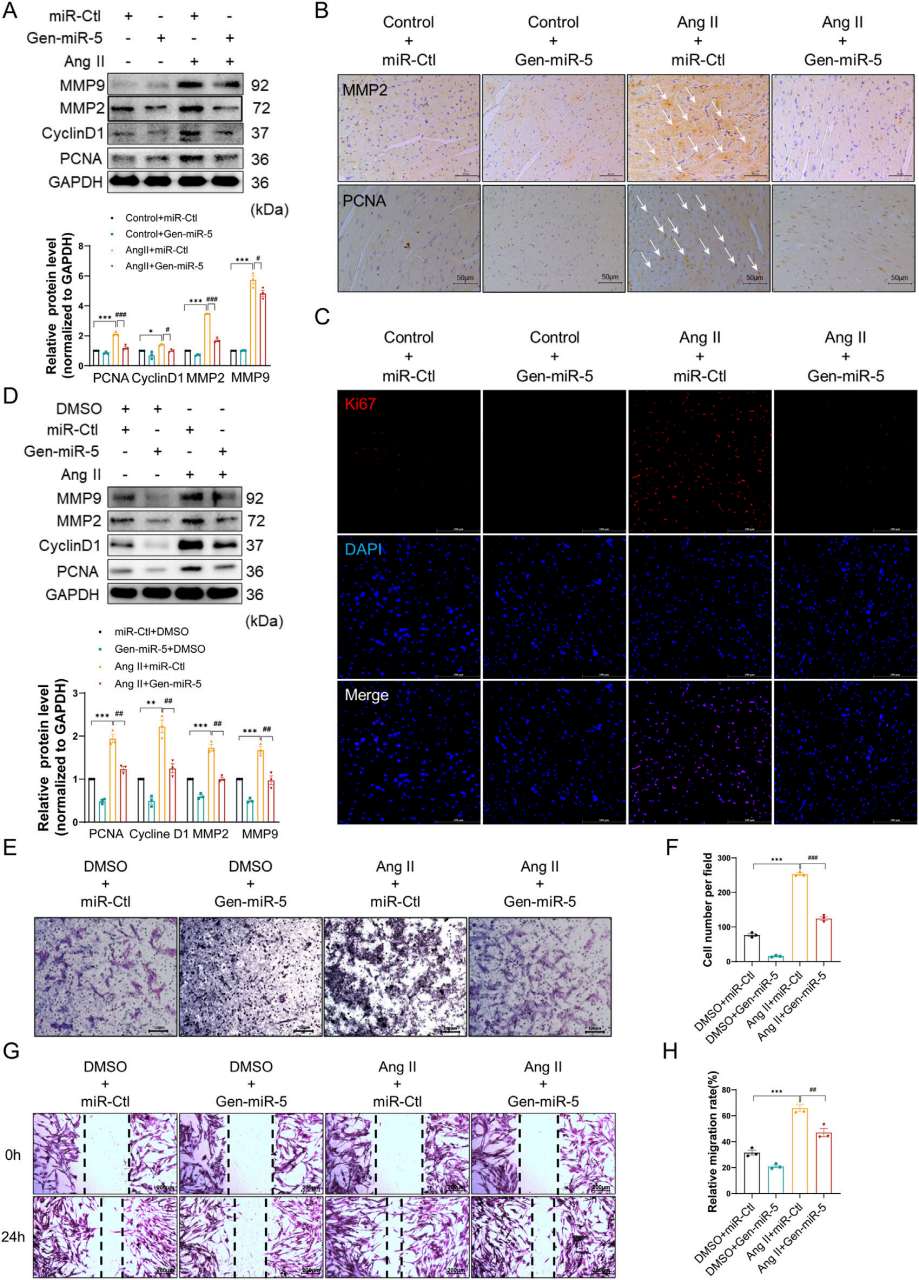
Gen-miR-5 inhibits Ang II-induced CF proliferation and migration **(A)** Protein expression levels of MMP9, MMP2, CyclinD1, and PCNA in cardiac tissue were assessed using Western blot (n = 3). **(B)** Immunohistochemical staining was used to assess the expression of MMP2 and PCNA in cardiac tissue. Scale bar = 50 μm. **(C)** Immunofluorescence staining was used to assess the expression of Ki67 in cardiac tissue. Red fluorescence indicates Ki67 and blue fluorescence marks the nuclei. Scale bar = 50 μm. **(D)** Protein expression levels of MMP9, MMP2, CyclinD1, and PCNA in cardiac fibroblasts were assessed using Western blot (n = 3). **(E)** Transwell assay used to assess the invasiveness of cardiac fibroblasts. Scale bar = 100 μm. **(F)** Quantification of cell invasion rate. **(G)** Wound healing assay to assess cardiac fibroblast migration. Scale bar = 200 μm. **(H)** Quantification of cell migration rate. Values are expressed as mean ± SEM. ^*^ *P* < 0.05, ^**^ *P* < 0.01, ^***^ *P* < 0.001, ^#^ *P* < 0.05, ^##^ *P* < 0.01, ^###^ *P* < 0.001 compared to the corresponding control group.

Activated CFs contribute to MF through their proliferation and migration to the site of injury. To investigate the effects of Gen-miR-5 on the biological behavior of CFs, we assessed the expression level of cellular migration markers MMP9 and MMP2, and proliferation protein Cyclin D1 and PCNA in cardiac tissues. The results indicated that Ang II increased the protein level of MMP9, MMP2, Cyclin D1, and PCNA. However, treatment with Gen-miR-5 significantly inhibited the proliferation and migration of CFs in cardiac tissues (Figure 4A). Similarly, the immunohistochemical staining results for MMP2 and PCNA were consistent with the Western blot findings (Figure 4B). To further confirm the effects of Gen-miR-5 on CFs proliferation, immunofluorescence staining for Ki67 expression was performed. The results showed that Ang II increased Ki67 expression in cardiac tissues, whereas Gen-miR-5 significantly reduced it (Figure 4C). Additionally, after transfecting CFs with Gen-miR-5 and treating them with Ang II, we assessed the expression of proliferation and migration protein. Gen-miR-5 significantly inhibited Ang II-induced expression of MMP9, MMP2, Cyclin D1, and PCNA (Figure 4D). Transwell and wound healing assays demonstrated that Gen-miR-5 also largely inhibited the promoting effect of Ang II-induced CFs migration (Figures 4E–H). These findings indicate that Gen-miR-5 significantly inhibits both the proliferation and migration of CFs.

### Gen-miR-5 specifically inhibits the expression of PFKP induced by Ang II

**FIGURE 5:**
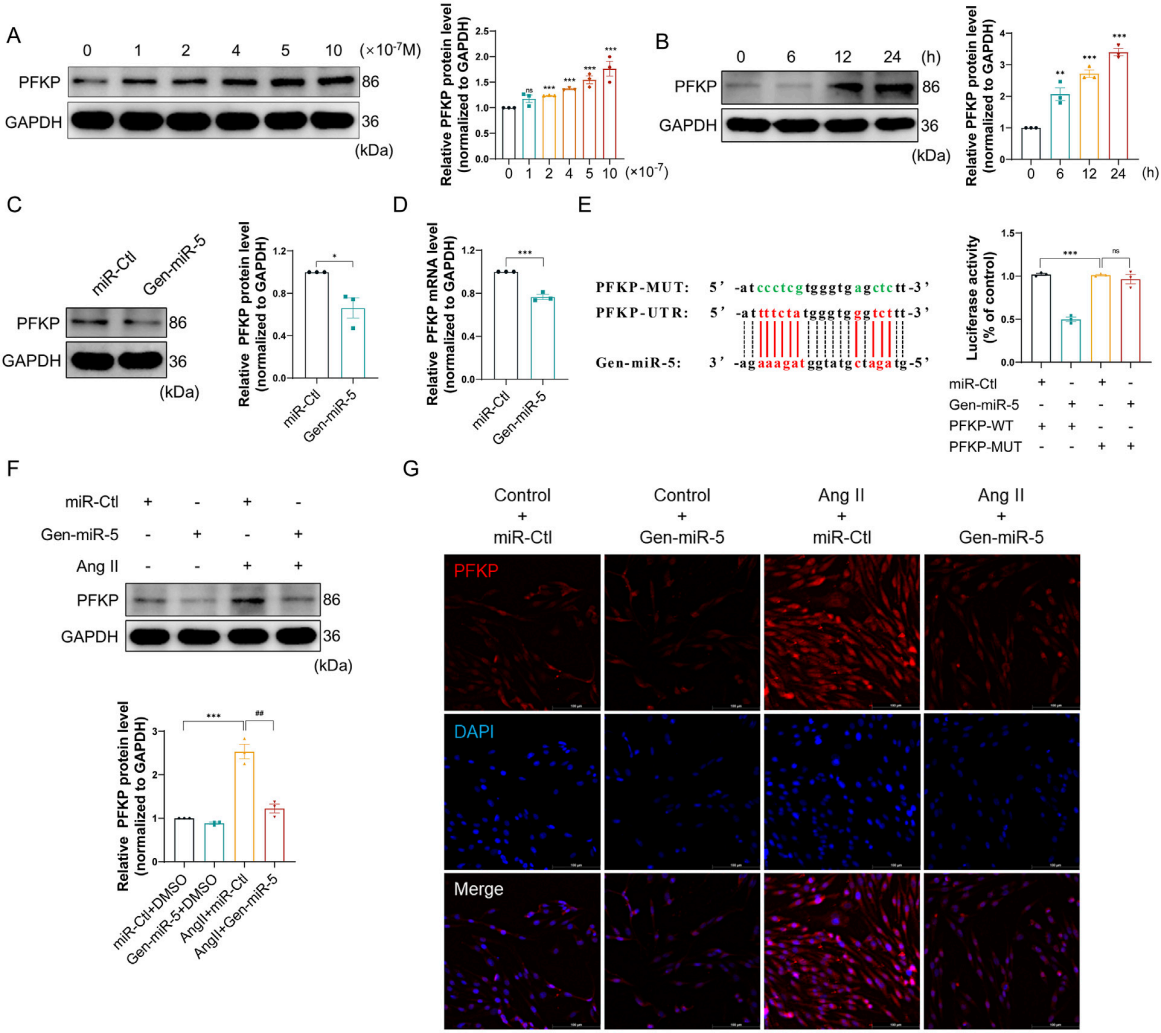
Gen-miR-5 can inhibit the expression of PFKP induced by Ang II **(A)** After treating cardiac fibroblasts with varying doses of Ang II for 24 h, PFKP protein expression levels were assessed using Western blot (n = 3). **(B)** Cardiac fibroblasts were treated with Ang II (1 μM) at specified times, and PFKP protein expression levels were assessed using Western blot (n = 3). **(C)** PFKP protein expression levels in cardiac fibroblasts were assessed using Western blot (n = 3). **(D)** PFKP mRNA levels in cardiac fibroblasts were measured using qRT-PCR (n = 3). **(E)** Dual-luciferase reporter assay was used to determine the binding interaction between PFKP and Gen-miR-5 in cardiac fibroblasts. The binding sites of Gen-miR-5 on the 3′ UTR of PFKP mRNA are marked in red, while the mutation sites are marked in green. **(F)** PFKP protein expression levels in cardiac fibroblasts were assessed using Western blot (n = 3). **(G)** Immunofluorescence staining was used to assess the expression of PFKP in cardiac fibroblasts. Red fluorescence indicates PFKP and blue fluorescence marks the nuclei. Scale bar = 100 μm. Values are expressed as mean ± SEM. ^*^ *P* < 0.05, ^**^ *P* < 0.01, ^***^ *P* < 0.001 compared to the corresponding control group. ^##^ *P* < 0.01 compared to the corresponding Ang II + miR-Ctl group.

Increasing research confirms the gene-regulatory role of plant-derived miRNAs in mammals. Through predictive analysis of Gen-miR-5’s target sequences, we identified a putative binding site within the 3′ UTR of PFKP. Initially, we examined the expression changes of PFKP in CFs. Treating CFs with varying doses of Ang II resulted in a dose-dependent increase in PFKP protein expression (Figure 5A). Subsequently, treating CFs at different time points with Ang II significantly increased PFKP protein expression in a time-dependent manner (Figure 5B). The results suggest a pivotal role for PFKP in the activation of CFs. In addition, Gen-miR-5 can effectively inhibit Ang II-induced CFs activation. Based on this, we put forward the hypothesis that Gen-miR-5 may inhibit CFs activation by inhibiting PFKP. We found that Gen-miR-5 inhibited PFKP expression at both protein and mRNA levels (Figures 5C,D). To confirm the binding site between PFKP 3′ UTR and Gen-miR-5, we constructed the luciferase reporter gene wild type and mutant plasmid of the PFKP 3′ UTR and co-transfected it with Gen-miR-5 into CFs for a dual-luciferase reporter assay. The results demonstrated a significant reduction in luciferase activity in the wild-type PFKP 3′ UTR group co-transfected with Gen-miR-5, whereas no significant change was observed in the mutant group (Figure 5E). Next, CFs were treated with Ang II and transfected with Gen-miR-5 to observe the expression of PFKP. The results showed that Gen-miR-5 effectively mitigated the Ang II-induced upregulation of PFKP expression (Figure 5F). Immunofluorescence staining further verified the inhibitory effect of Gen-miR-5 on PFKP during CF activation (Figure 5G). These findings suggest that Gen-miR-5 suppresses Ang II-induced PFKP expression by targeting the 3′ UTR of the PFKP gene.

### PFKP downregulation by Gen-miR-5 attenuates the proliferation and migration of CFs

**FIGURE 6:**
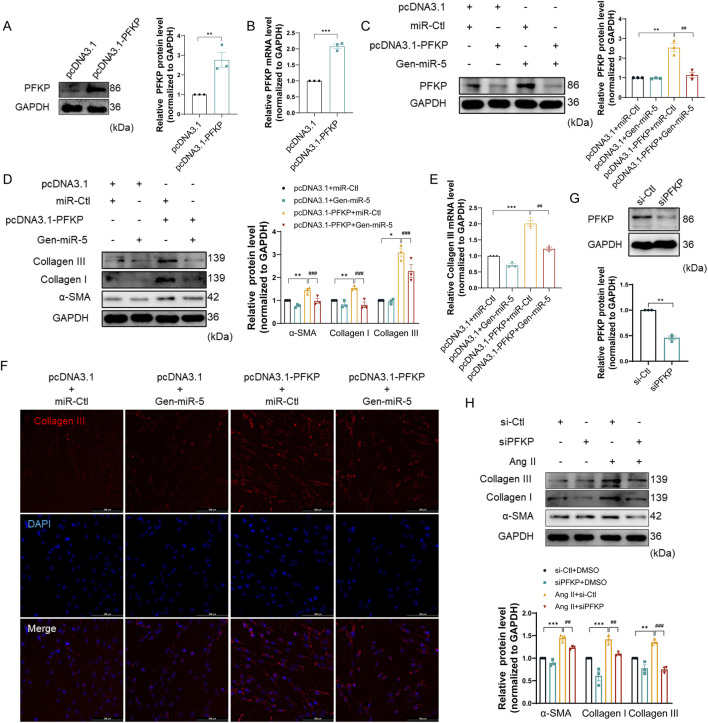
Gen-miR-5 targets PFKP to inhibit the proliferation and migration of CFs **(A)** PFKP protein expression levels in cardiac fibroblasts were assessed using Western blot (n = 3). **(B)** PFKP mRNA levels in cardiac fibroblasts were measured using qRT-PCR (n = 3). **(C)** PFKP protein expression levels in cardiac fibroblasts were assessed using Western blot (n = 3). **(D)** Protein expression of Collagen III, Collagen I, and α-SMA in cardiac fibroblasts was detected by Western blot (n = 3). **(E)** Collagen III mRNA levels in cardiac fibroblasts were detected by qRT-PCR (n = 3). **(F)** Collagen III expression in cardiac fibroblasts was detected by immunofluorescence staining. Red fluorescence is Collagen III and blue fluorescence is the nucleus. **(G)** PFKP protein expression levels in cardiac fibroblasts were assessed using Western blot (n = 3). **(H)** Protein expression of Collagen III, Collagen I, and α-SMA in cardiac fibroblasts was detected by Western blot (n = 3). Values are expressed as mean ± SEM. *P < 0.05, **P < 0.01, ***P < 0.001, ##*P* < 0.01,###*P* < 0.001 compared to the corresponding control group.

PFKP, a key enzyme in the glycolytic process, has not yet been reported in the pathogenesis of MF. To further elucidate the specific mechanisms of PFKP in MF, we transfected pcDNA3.1-PFKP plasmids into CFs to achieve overexpression of PFKP (Figures 6A,B). Moreover, when CFs were transfected with pcDNA3.1-PFKP and Gen-miR-5, the upregulation of PFKP by overexpression plasmid was greatly abolished. (Figure 6C). Subsequently, the effects of PFKP on CFs activation and collagen synthesis were evaluated by assessing the expression levels of Collagen III, Collagen I, and α-SMA. We showed that overexpression of PFKP significantly enhanced the expression levels of Collagen III, Collagen I, and α-SMA, while Gen-miR-5 effectively suppressed the expression of all three (Figures 6D,E). Immunofluorescence staining experiments yielded consistent results (Figure 6F). To further confirm the role of PFKP in CF activation, we transfected CFs with PFKP-siRNA and evaluated the knockdown efficiency of PFKP. The results showed that siPFKP727 reduced PFKP expression by 40%–55% (Figure 6G; Supplementary Figure S1). To further confirm the role of PFKP in CFs activation, we transfected CFs with PFKP-siRNA and assessed the effects of Ang II on the expression of Collagen III, Collagen I, and α-SMA. The results indicated that the knockdown of PFKP effectively blocked the induction of Collagen III, Collagen I, and α-SMA protein expression by Ang II (Figure 6H). The above results confirm that PFKP plays a critical role in CFs activation, while Gen-miR-5 inhibits PFKP-induced CFs activation and collagen synthesis.

### PFKP synergistically induces CFs proliferation and migration in conjunction with lactate

**FIGURE 7:**
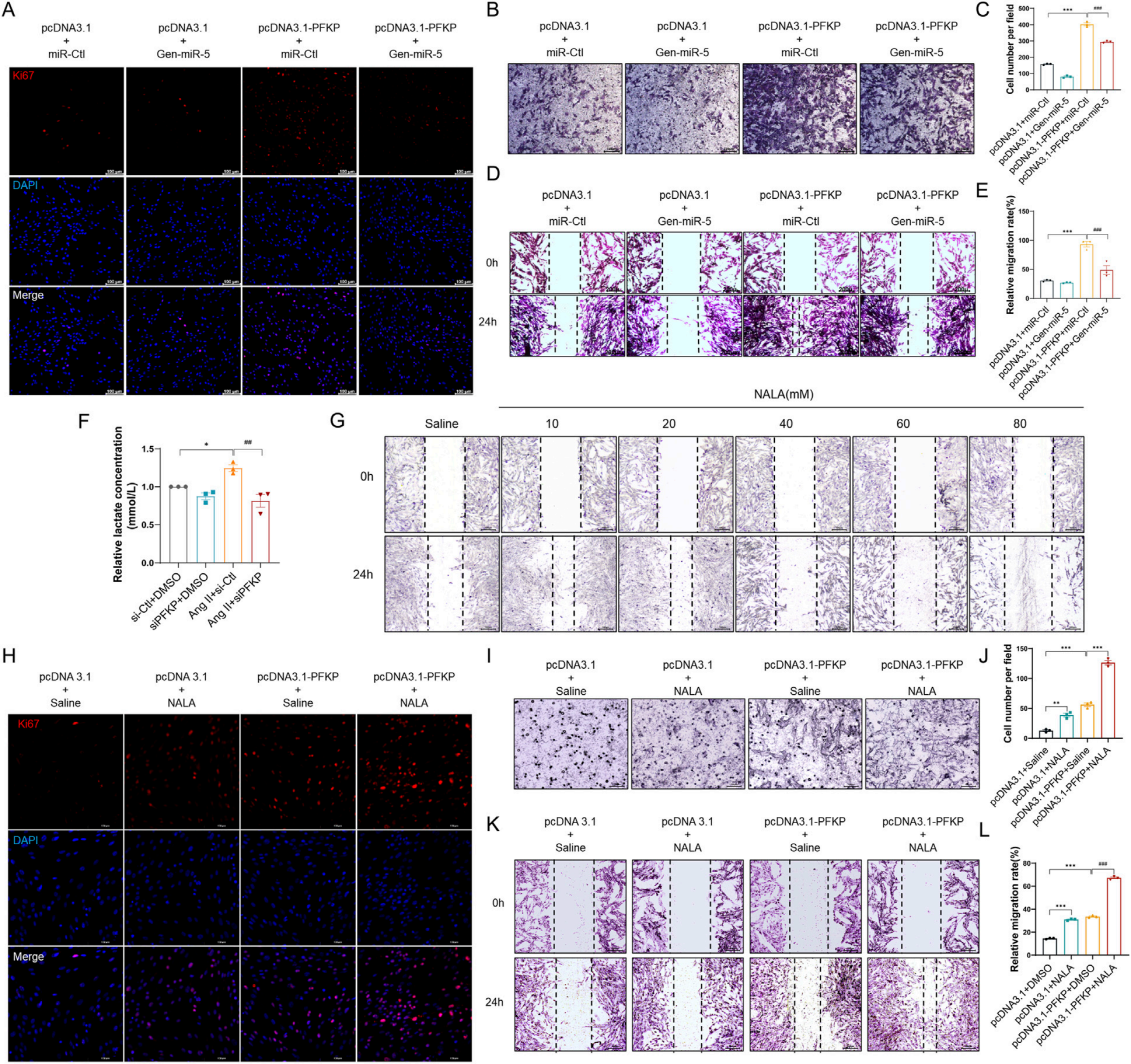
PFKP synergistically induces CF proliferation and migration in conjunction with lactate **(A)** Ki67 expression in cardiac fibroblasts was detected by immunofluorescence staining. Red fluorescence is Ki67 and blue fluorescence is nucleus. Scale bar = 100 μm. **(B)** Transwell detection of myocardial fibroblast invasion. Scale bar = 100 μm. **(C)** Quantification of cell invasion rate. **(D)** Cell wound healing assay to detect myocardial fibroblast migration. Scale bar = 200 μm. **(E)** Quantification of cell migration rate. **(F)** Changes in lactate levels. **(G)** Wound healing assay to assess cardiac fibroblast migration. Scale bar = 200 μm. **(H)** Ki67 expression in cardiac fibroblasts was detected by immunofluorescence staining. Red fluorescence is Ki67 and blue fluorescence is nucleus. Scale bar = 100 μm. **(I)** Transwell detection of myocardial fibroblast invasion. Scale bar = 100 μm. **(J)** Quantification of cell invasion rate. **(K)** Cell wound healing assay to detect myocardial fibroblast migration. Scale bar = 200 μm. **(L)** Quantification of cell migration rate. Values are expressed as mean ± SEM. ***P < 0.001, ###*P* < 0.001 compared to the corresponding control group.

Previous experiments have confirmed that PFKP plays a crucial role in CFs activation. However, it remains unclear how PFKP exerts its role in promoting MF by affecting CFs proliferation and migration. Research indicates that glycolysis plays a crucial role in the reprogramming of CFs (Ding et al., 2022). Given the metabolic demands during CFs activation, glycolysis provides an effective metabolic pathway. PFKP is known to accelerate glycolysis and promote lactate accumulation, which may be an important contributor to fibrosis. First, we performed Ki67 immunofluorescence staining on CFs. The results revealed that overexpression of PFKP promoted CFs proliferation, which could be largely reversed by transfecting Gen-miR-5 (Figure 7A). Transwell assays demonstrated that Gen-miR-5 significantly inhibited the invasion of CFs promoted by the overexpression of PFKP (Figures 7B,C). Additionally, the wound healing assay indicated that overexpression of PFKP significantly enhanced CFs migration, while Gen-miR-5 effectively inhibited this pathological change (Figures 7D,E). As one of the key products of glycolysis, lactate serves as a substrate for cardiac energy supply. To further investigate the effect of PFKP on lactate production in CFs, we knocked down PFKP in Ang II-treated CFs (Supplementary Figure S1). The results showed that knocking down PFKP reduced Ang II-induced lactate production in CFs (Figure 7F). Using wound healing assay, we found that NALA at 20 mM significantly promotes the migration of CFs (Figure 7G). To investigate the role of PFKP and the glycolytic product lactate in the fibrosis process, NALA was added following PFKP overexpression in CFs to observe changes in cell proliferation. The results showed that both NALA and PFKP overexpression promote the proliferation of CFs. Moreover, a synergistic effect was observed between NALA and PFKP, with their combined application significantly enhancing the pro-proliferative effect (Figure 7H). Additionally, we investigated the effects of NALA and PFKP on the invasive capability of CFs using Transwell assays. The results demonstrated that NALA and PFKP synergistically enhance the invasive capacity of CFs (Figures 7I,J). The results of the wound healing assay indicated that both PFKP overexpression and lactate promote CFs migration, and their combined use leads to a cumulative effect, further enhancing cell migration (Figures 7K,L). Those results indicated that Gen-miR-5 promoted the proliferation and migration of CFs through targeted inhibition of PFKP, and PFKP could synergistically promote CFs proliferation and migration with lactate.

## Discussion

Heart failure represents the common end-stage outcome of most cardiovascular diseases, with cardiac fibrosis serving as the underlying pathological mechanism that has emerged as a critical challenge in cardiovascular medicine (Al Hattab and Czubryt, 2017). MF is characterized by disrupted extracellular matrix homeostasis, leading to excessive collagen deposition, structural damage to the heart, impaired cardiac function, and accelerated progression toward heart failure (van den Borne et al., 2010). At the core of these pathological changes lies the persistent activation and abnormal proliferation/migration of cardiac fibroblasts, which play a pivotal role in driving fibrotic progression.

“Heat Toxin Theory” of cardiac diseases has been widely validated in clinical and experimental studies. It posits that MF results from the accumulation of heat toxins, phlegm, and blood stasis and that methods of heat-clearing and detoxification can effectively prevent and treat cardiovascular diseases. For decades, research on traditional Chinese medicine (TCM) has predominantly centered on elucidating the mechanisms of action through the study of chemical constituents. However, in most cases, a single compound fails to replicate the therapeutic efficacy of TCM decoctions, and the identification of active ingredients remains challenging. In recent years, miRNAs derived from Chinese medicinal herbs have emerged as novel active molecules, with their disease-modulating mechanisms increasingly elucidated (Ji et al., 2019; Li et al., 2019). Unlike conventional phytochemicals, plant-derived miRNAs exhibit superior targeting specificity and regulatory precision, exerting therapeutic effects through gene expression modulation. Advancing research has revealed that even after rigorous processing, TCM materials retain structurally stable and functionally abundant miRNAs, suggesting their potential role as key active components (Huang et al., 2019). Moreover, the ability of TCM-derived miRNAs to cross-kingdom regulate gene expression opens new avenues for the development of RNA therapeutics.

*Gentianella acuta*, commonly used in Mongolian and Evenki folk medicine, is known for its heat-clearing and detoxification effects. Local hunters use it to treat cardiovascular diseases such as angina and arrhythmia, with notably effective results. Based on the current extensive research on plant-derived miRNAs regulating mammalian gene expression in a cross-kingdom manner (Zhang et al., 2023), we screened and identified six unique miRNAs from *G. acuta* by high-throughput sequencing. This study found that Gen-miR-5 can be absorbed into the bloodstream and accumulates in the heart, reaching peak enrichment levels 6 h after administration and being completely degraded after 48 h, with no toxic side effects on the liver or kidneys. Furthermore, in a mouse model of cardiac fibrosis induced by Ang II-mediated pressure overload, Gen-miR-5 was observed to significantly suppress excessive collagen fiber deposition and improve cardiac dysfunction. Therefore, we propose that Gen-miR-5, as a novel active component of *G. acuta* in treating cardiovascular diseases, can cross-kingdom regulate to exert inhibit myocardial fibrosis effects in mice. These findings provide new evidence supporting the cardiovascular protective role of *G. acuta*. Whether Gen-miR-5 exerts its anti-MF effects by suppressing the proliferation and migration of CFs is currently unknown. Our findings demonstrate that Ang II induces time- and concentration-dependent activation of CFs, leading to their pathological proliferation and migration. Notably, Gen-miR-5 treatment effectively attenuated Ang II-triggered CFs activation and its downstream pathological responses, including excessive proliferation and migration. Those findings suggest that Gen-miR-5 may alleviate myocardial fibrosis by suppressing the excessive proliferation and migration of CFs. It should be noted that current understanding of how TCM-derived miRNAs maintain their structural stability and bioavailability *in vivo* remains inadequate, and further investigations are warranted to determine their minimum effective concentration in biological systems. Moreover, greater attention should be paid to potential structural modifications of these exogenous miRNAs under physiological conditions as well as their off-target effects.

During MF, fibroblasts undergo metabolic reprogramming from oxidative phosphorylation to a glycolysis-dependent metabolic mode (Ji et al., 2022; Zhang et al., 2024). Inhibiting the increase in glycolysis rate and reducing lactate accumulation can mitigate the progression of MF (Chen et al., 2021). PFKP, a key rate-limiting enzyme in glycolysis, plays a crucial role in metabolic remodeling (Fan et al., 2021). It has been reported that inhibiting PFKP can downregulate BNP expression and protein synthesis during myocardial hypertrophy, thereby alleviating cardiac remodeling (Vigil Garcia et al., 2021; Wu et al., 2024). This latest research suggests that PFKP has significant potential research value in cardiovascular diseases. However, the role of PFKP in fibroblasts remains unclear, making it crucial to fill this gap. Our research has found that Ang II-induced MF upregulates PFKP expression both in the heart and in CFs, and the high expression of PFKP promotes the glycolytic pathway and accelerates the accumulation of lactate. Meanwhile, overexpression of PFKP in cardiac myofibroblasts can increase cell proliferation, and migration and enhance collagen deposition. Transfection with Gen-miR-5 and knockdown of PFKP both alleviate these pathological changes and inhibit the occurrence of MF. We confirmed that Gen-miR-5 has a binding site in the PFKP 3′ UTR, and therefore, we propose that Gen-miR-5 improves MF by inhibiting PFKP expression. Therefore, we believe that Gen-miR-5 improves MF by suppressing the expression of PFKP. Lactate, a by-product of glycolysis, serves as a crucial energy source for mitochondrial respiration (Murashige et al., 2020). It accumulates extensively during cardiac hypertrophy and heart failure (Cluntun et al., 2021), leading to impaired cardiac contractile function (Bertero and Maack, 2018). Our research shows that Ang II significantly increases lactate levels in CFs. In addition, exogenous treatment with NALA enhanced CFs proliferation, and migration and accelerated the development of MF. Conversely, knocking down PFKP inhibits glycolysis, reducing the production and accumulation of lactate and thereby improving MF. This study has found that PFKP plays a critical role in MF by regulating the biological behavior of CFs by controlling the production of lactate in glycolysis. As a key upstream regulatory enzyme in the glycolytic pathway, PFKP may exert extensive biological effects, with lactate modulation representing just one crucial aspect, which needs further investigation. The precise molecular mechanisms through which lactate regulates CFs excessive proliferation and migration remain to be fully elucidated. These knowledge gaps constitute critical research directions for establishing PFKP as a therapeutic target for cardiac fibrosis.

## Conclusion

In summary, PFKP serves as a key regulatory factor in the process of MF, participating in Ang II-induced fibrosis through the regulation of cellular metabolic reprogramming. Gen-miR-5 from *G. acuta*, by specifically inhibiting the expression of Ang II-induced PFKP, reduces lactate production, suppresses the activation of CFs, and improves cardiac function, proving to be an effective approach for treating MF. This study provides new and compelling evidence for the cross-kingdom therapeutic potential of plant-derived miRNAs.

## Data Availability

The original contributions presented in the study are included in the article/Supplementary Material, further inquiries can be directed to the corresponding authors.

## Supplementary material

SUPPLEMENTARY FIGURE S1 PFKP protein expression levels in cardiac fibroblasts were assessed using Western blot (n = 3). Values are expressed as mean Ã‚Â± SEM. *P 0.05Ã¯\x82\xBCÅ’**PÃ¯\x82\xBCÅ“0.01 compared to the corresponding control group.

## Abbreviations

CFs, cardiac fibroblasts; *G. acuta*, *Gentianella acuta*; TCM, traditional Chinese medicine; MF, myocardial fibrosis; Ang II, Angiotensin II; LVPW, left ventricular posterior wall thickness; H&E, hematoxylin and eosin; ECG, electrocardiogram; FISH, RNA fluorescence *in situ* hybridization.
