# Supplementary material for: Gen-miR-5 derived from Gentianella acuta inhibits PFKP to prevent fibroblast activation and alleviate myocardial fibrosis
Source: Front Pharmacol. 2025 May 2;16:1578877. doi: 10.3389/fphar.2025.1578877 (PMC12081263; doi:10.3389/fphar.2025.1578877)
Supplement: Supplementary file 1 [file Table1.docx]

**Table S1**

Primer sequences used for qRT-PCR experiments-Mouse

| Gene | Primer | Sequence (5’-3’) |
| --- | --- | --- |
| GAPDH | Forward | AGGTCGGTGTGAACGGATTTG |
|  | Reverse | TGTAGACCATGTAGTTGAGGTCA |
| Collagen Ⅰ | Forward | TGAACGTGGTGTACAAGGTC |
|  | Reverse | CCATCTTTACCAGGAGAACCAT |
| Collagen Ⅲ | Forward | GAAAGAATGGGGAGACTGGAC |
|  | Reverse | TACCAGGTATGCCTTGTAATCC |
| α-SMA | Forward | CGTGGCTATTCCTTCGTGACTACTG |
|  | Reverse | CGTCAGGCAGTTCGTAGCTCTTC |
| PFKP | Forward | CATGAATGCTGCTGTCCGTG |
|  | Reverse | CATGCCTTGGTAACCCTCGT |
| Gen-miR-5 | Forward | GCGCGTAGATCGTATGGTAGAAAGA |
| U6 | Forward | CGCTTAGGCAGCACATATAC |
|  | Reverse | TTCACGAATTTGCGTGTCATC |
