# Supplementary figures and images for: Gen-miR-5 derived from Gentianella acuta inhibits PFKP to prevent fibroblast activation and alleviate myocardial fibrosis
Source: Front Pharmacol. 2025 May 2;16:1578877. doi: 10.3389/fphar.2025.1578877 (PMC12081263; doi:10.3389/fphar.2025.1578877)

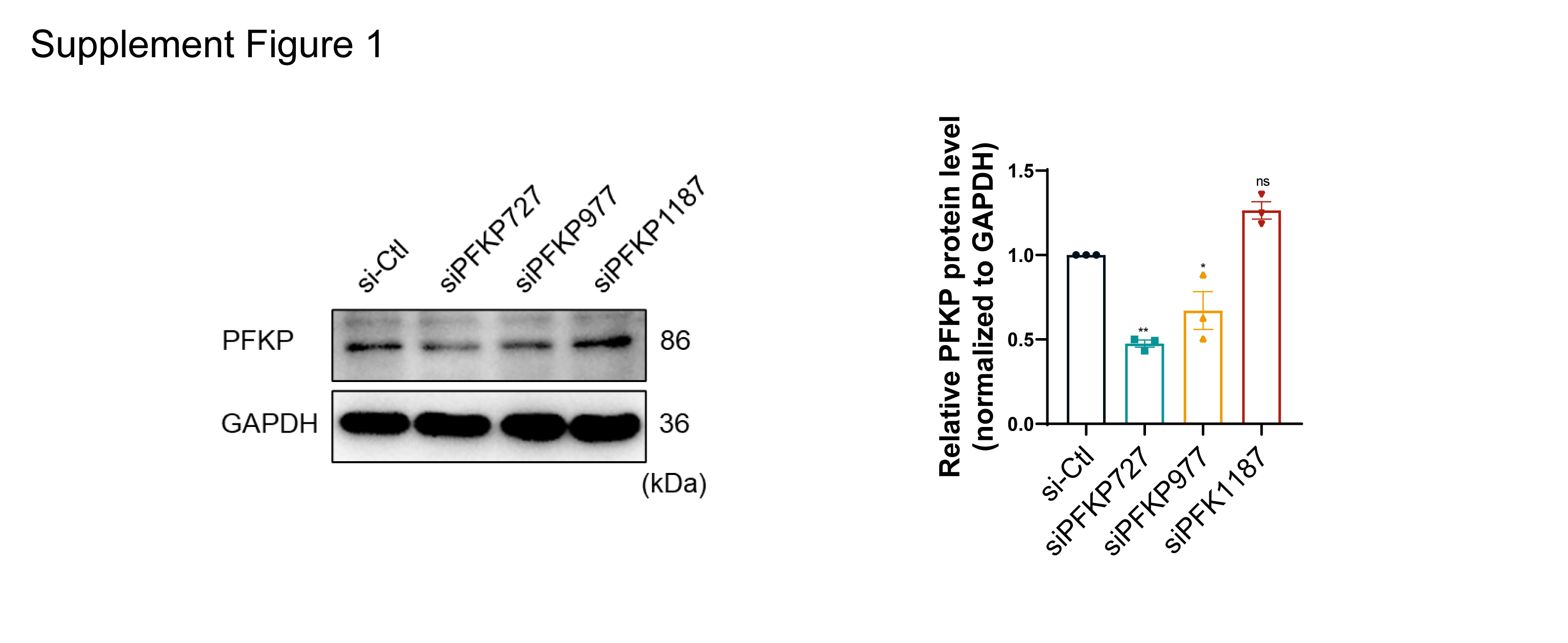

Supplement: Supplementary file 2 [file Image1.jpeg]
