# Supplementary material for: Gen-miR-5 derived from Gentianella acuta inhibits PFKP to prevent fibroblast activation and alleviate myocardial fibrosis
Source: Front Pharmacol. 2025 May 2;16:1578877. doi: 10.3389/fphar.2025.1578877 (PMC12081263; doi:10.3389/fphar.2025.1578877)

Fig.3 A

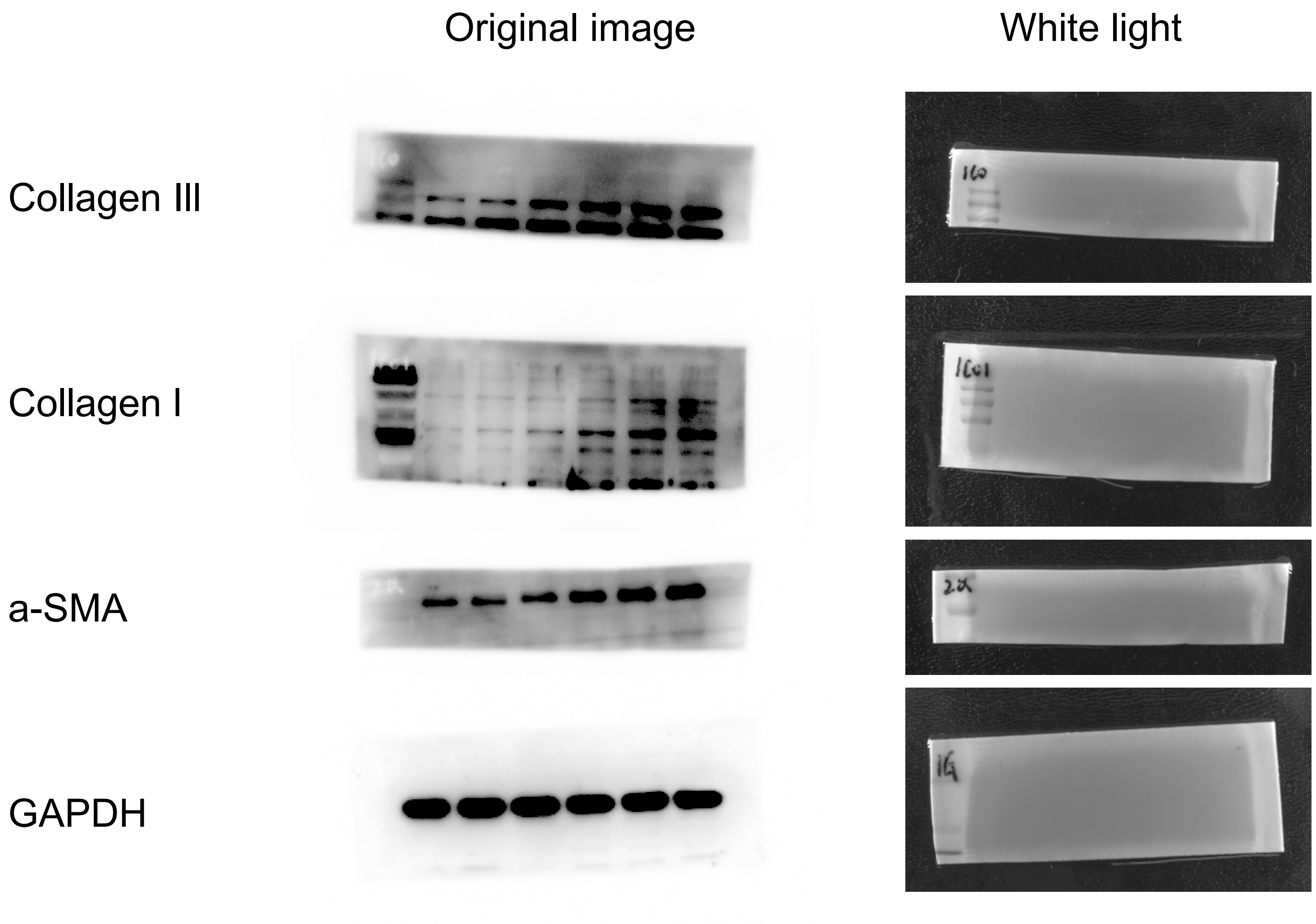
Original image
White light
Collagen III
Collagen I
a-SMA
GAPDH

Fig.3 B

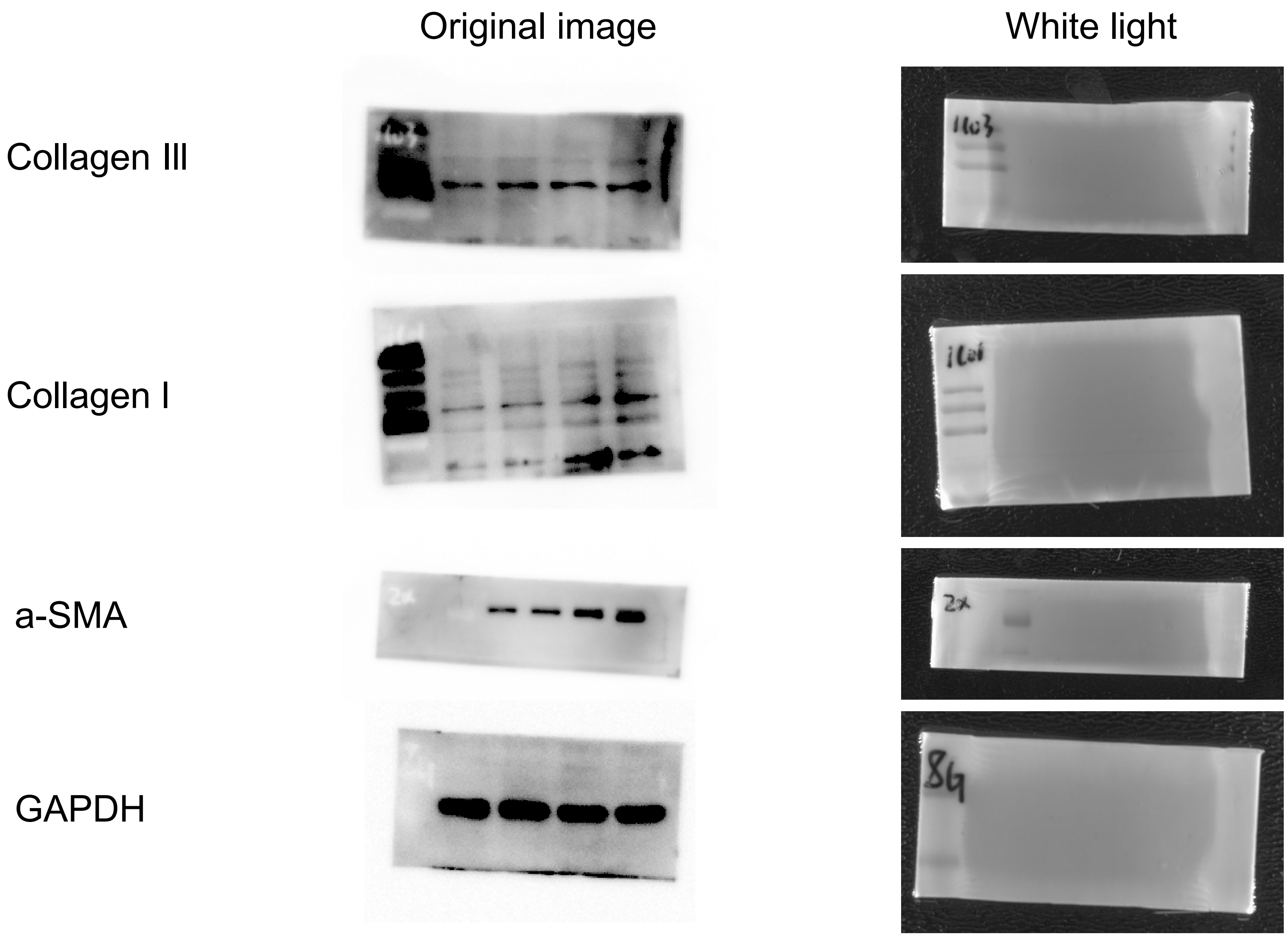
Original image
White light
Collagen III
Collagen I
a-SMA
GAPDH

Fig.3 C

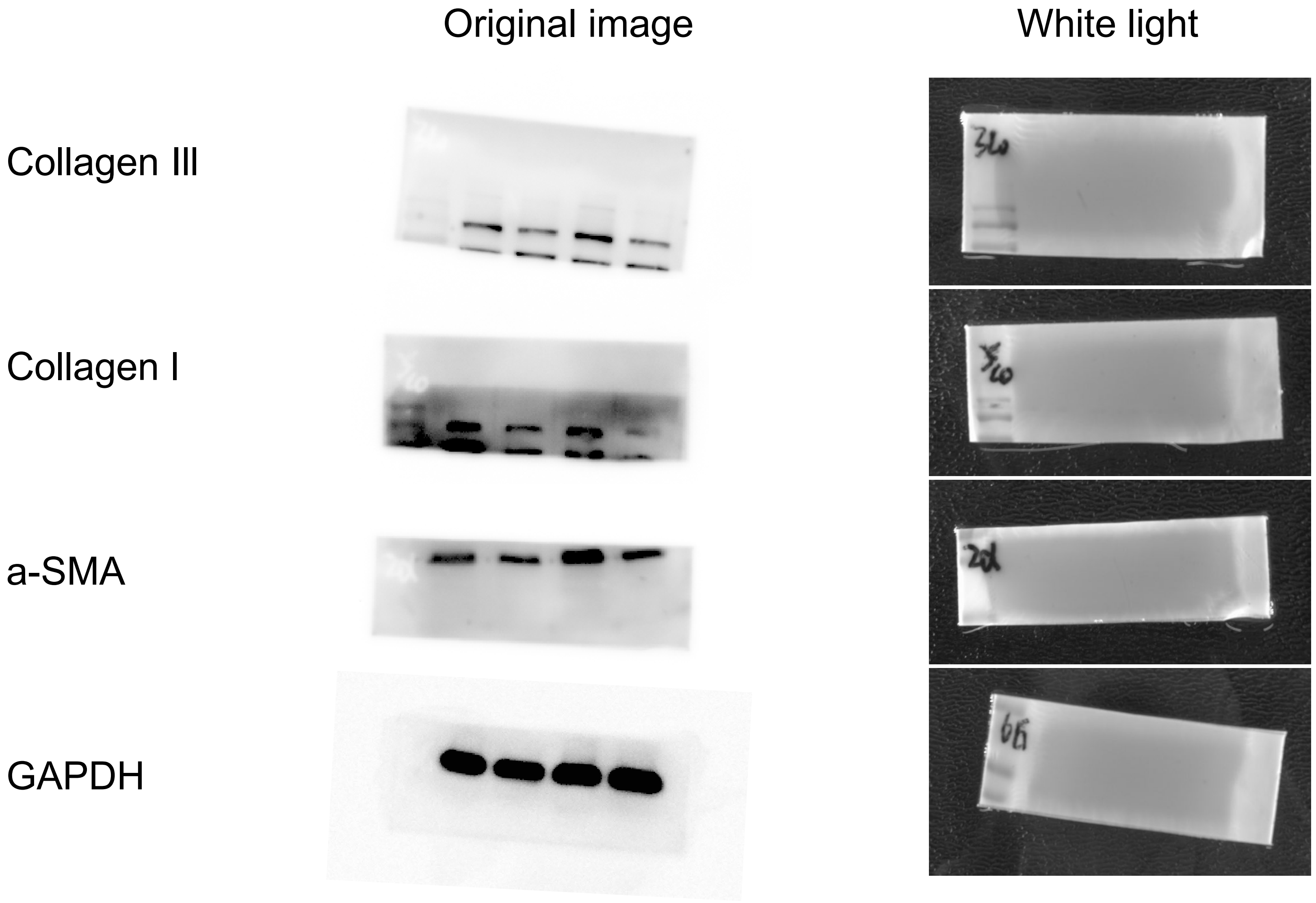
Original image
White light
Collagen III
Collagen I
a-SMA
GAPDH

Fig.3 D

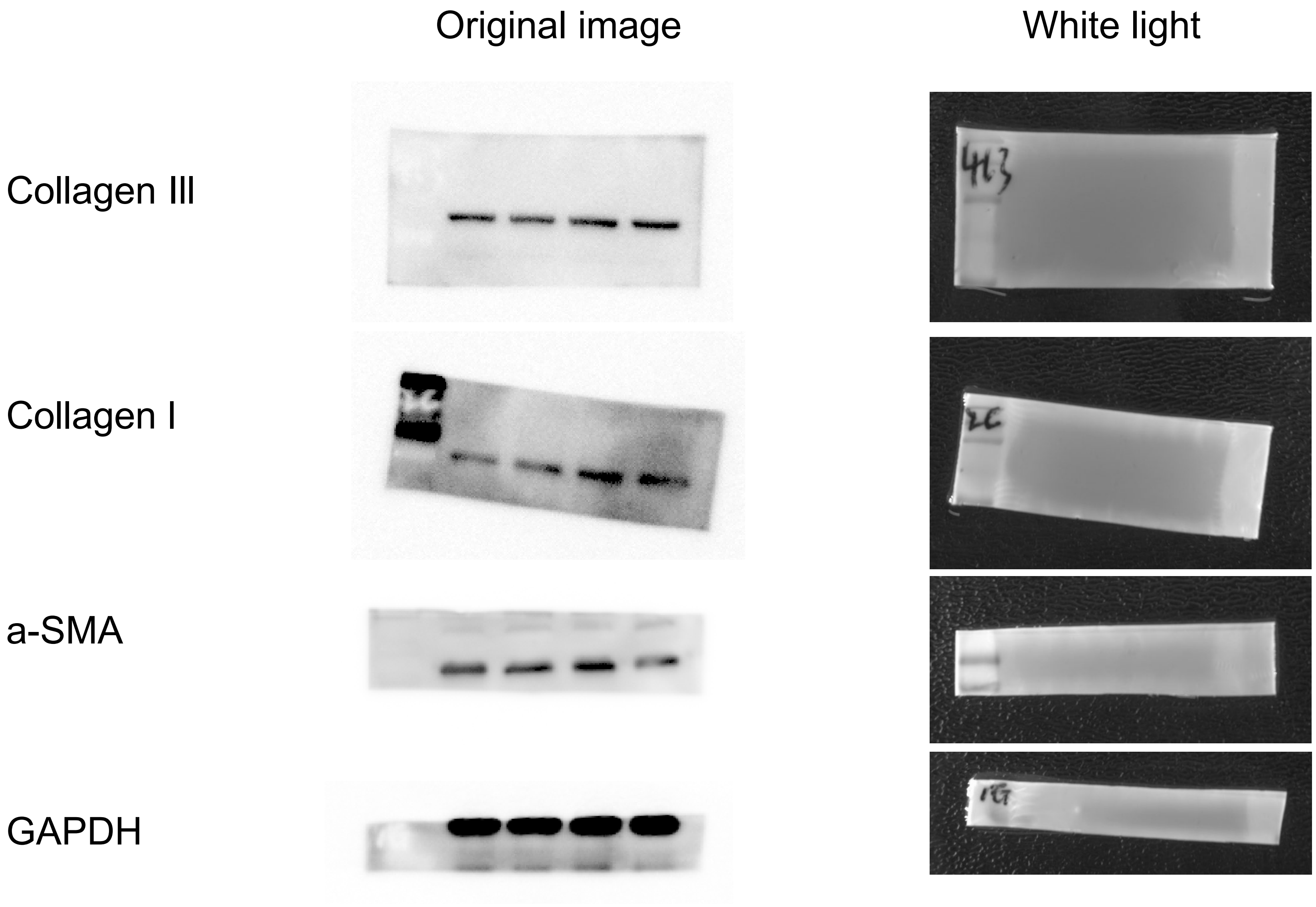
Original image
White light
Collagen III
Collagen I
a-SMA
GAPDH

Fig.4 A

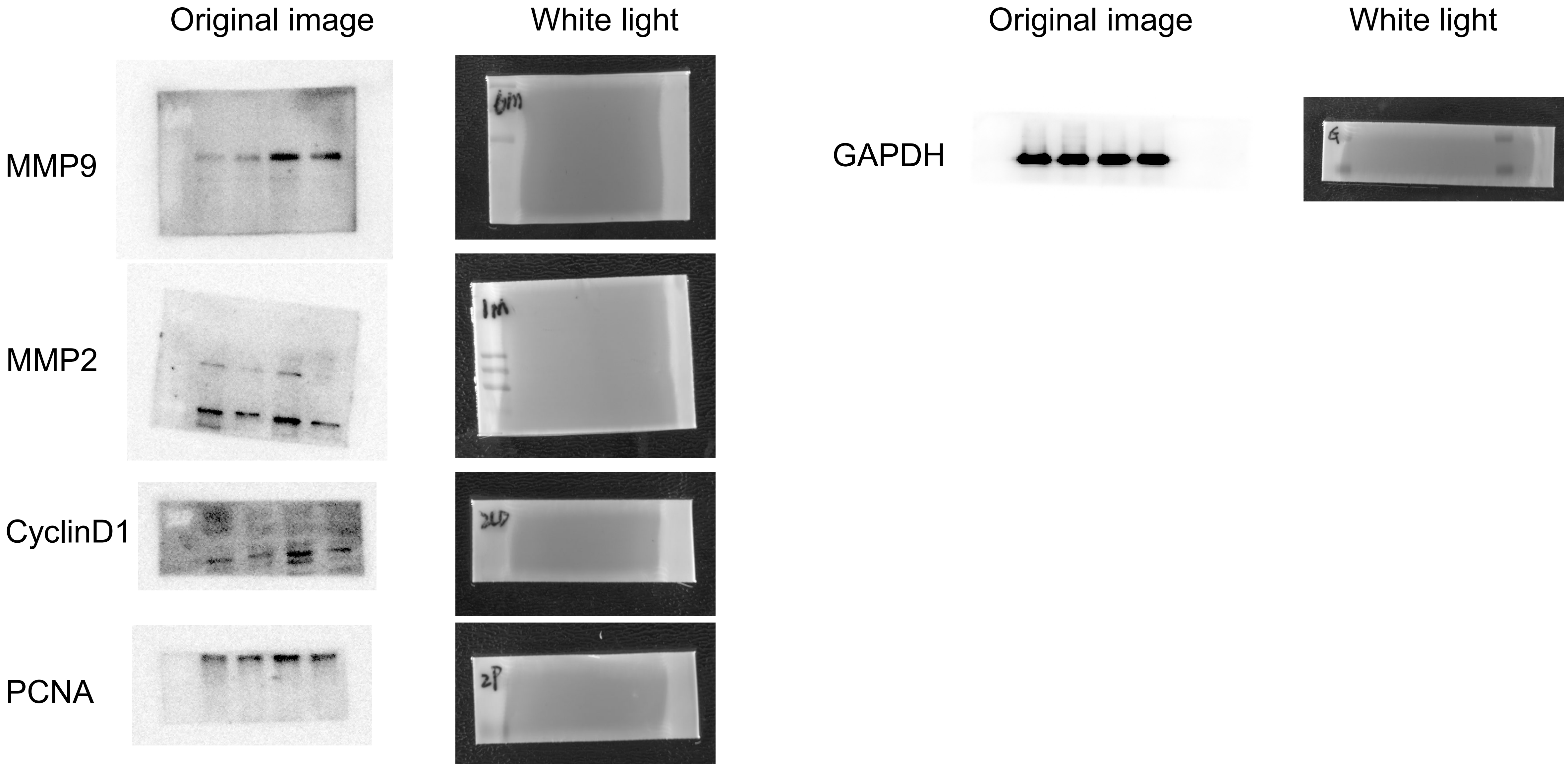
Original image
White light
Original image
White light
MMP9
MMP2
CyclinD1
PCNA
GAPDH

Fig.4 D

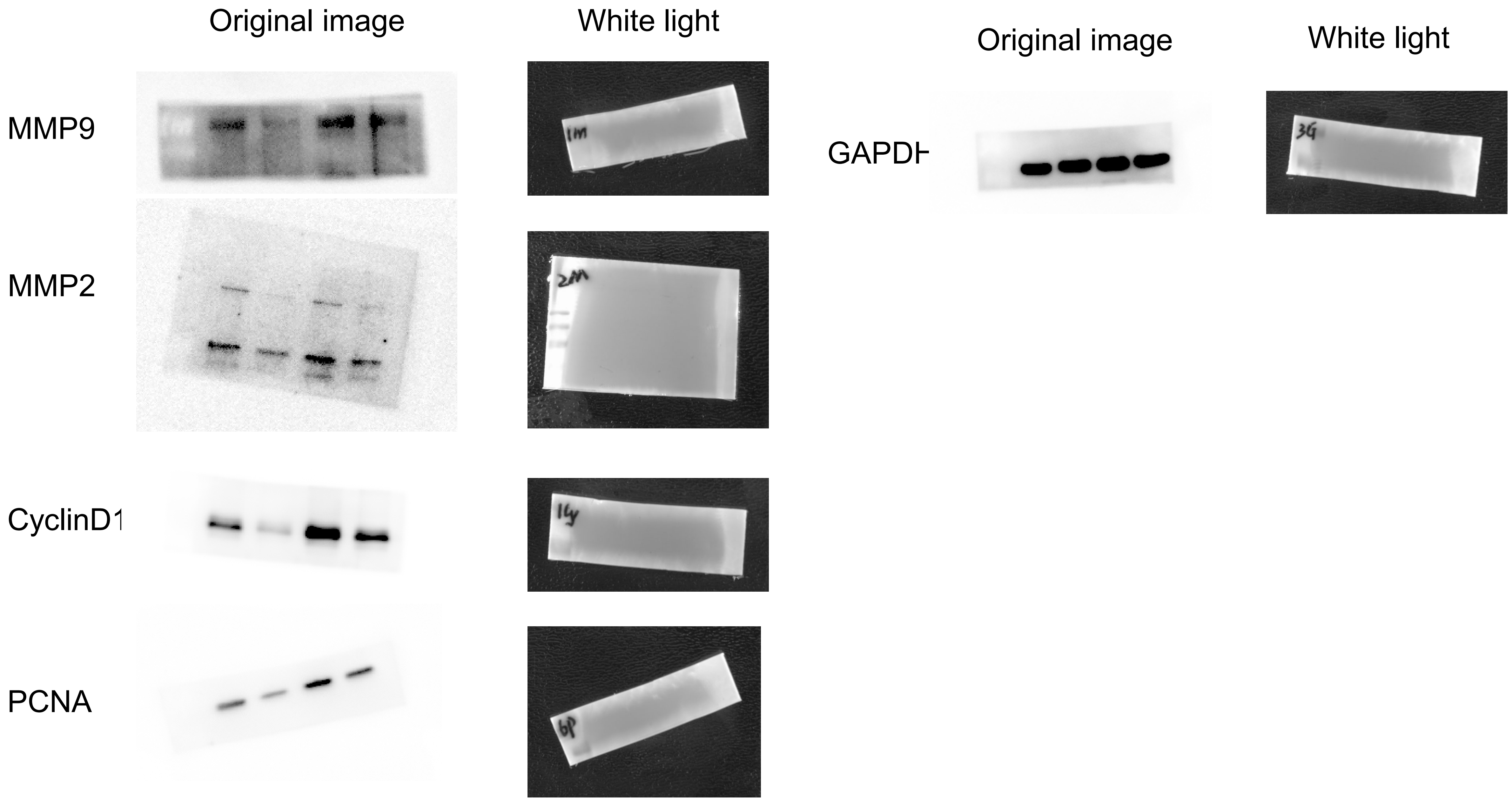
Original image
White light
Original image
White light
MMP9
MMP2
CyclinD
PCNA
GAPD

Fig.5 A

Original image

White light

PFKP

GAPDH

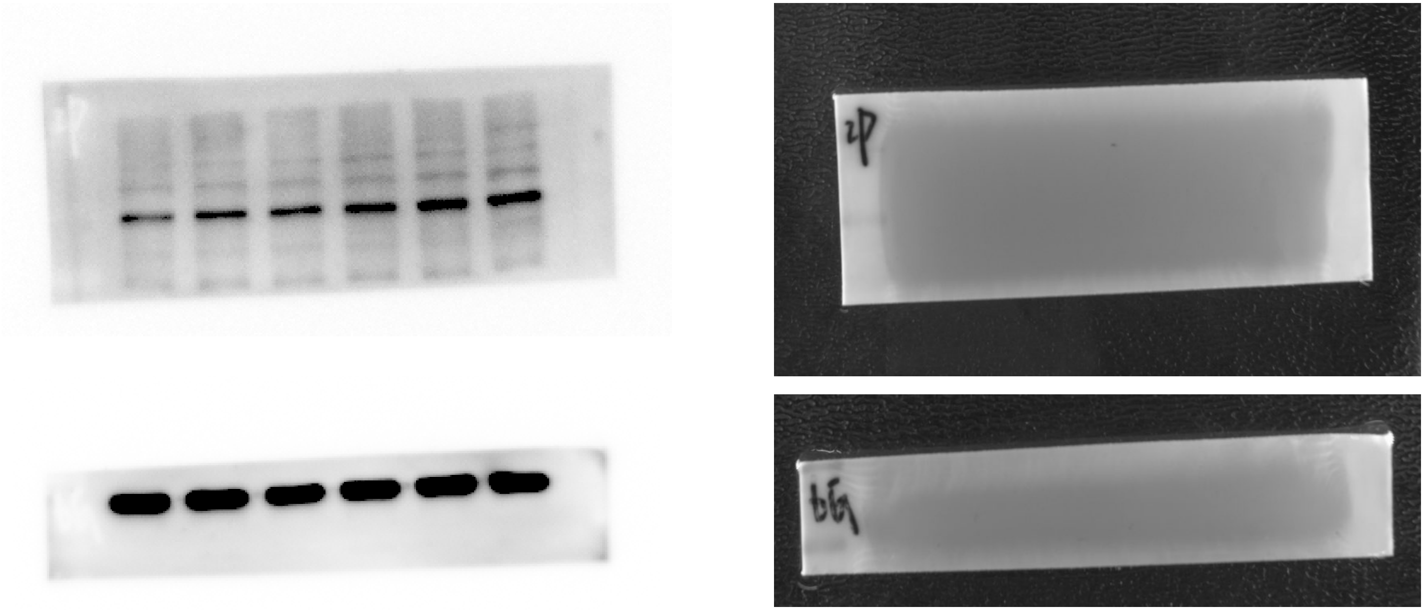

Fig.5 B

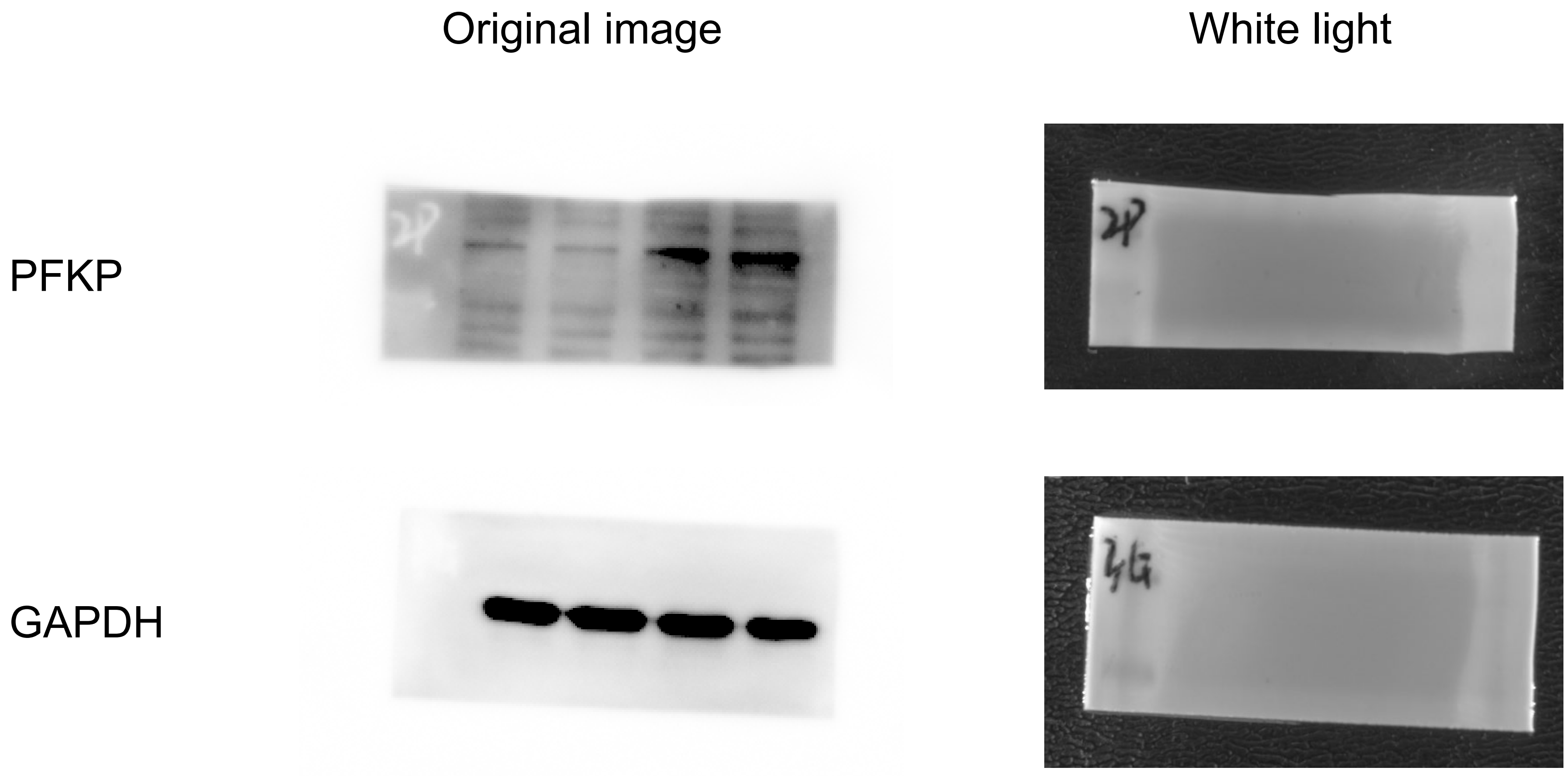

Fig.5 C

Original image

White light

PFKP

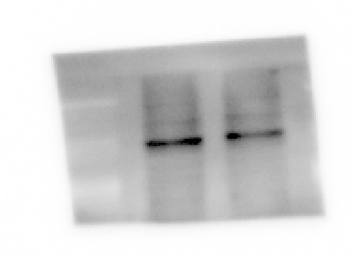

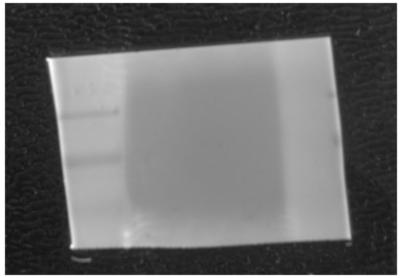

GAPDH

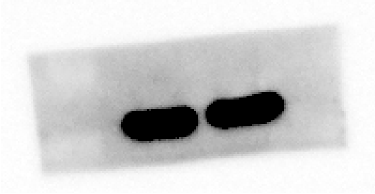

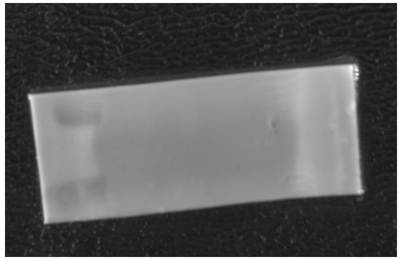

Fig.5 C

Original image White light

PFKP

GAPDH

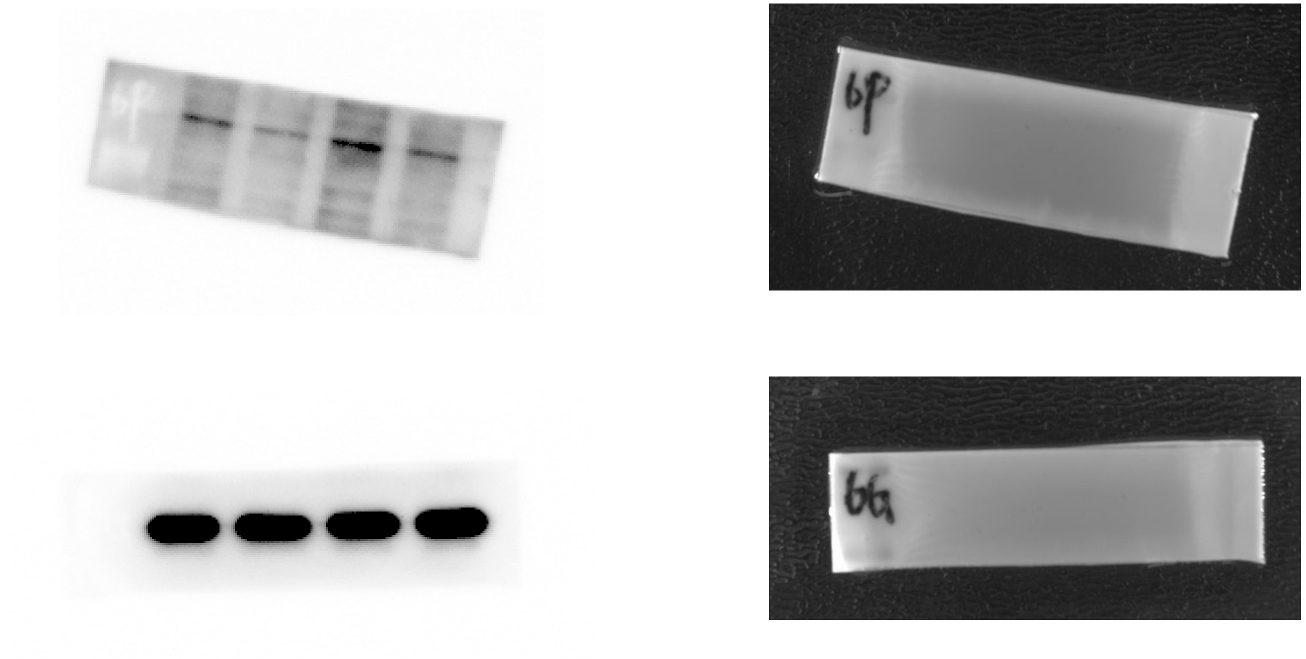

Fig.6 A

Original image | White light

PFKP

GAPDH

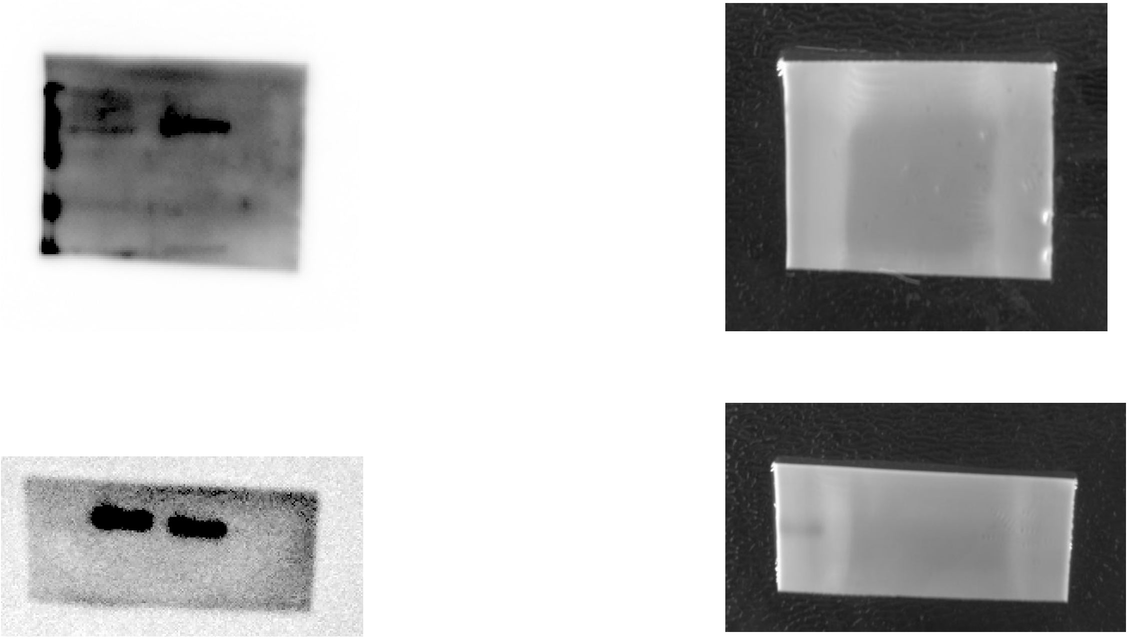

Fig.6 C

Original image

White light

PFKP

GAPDH

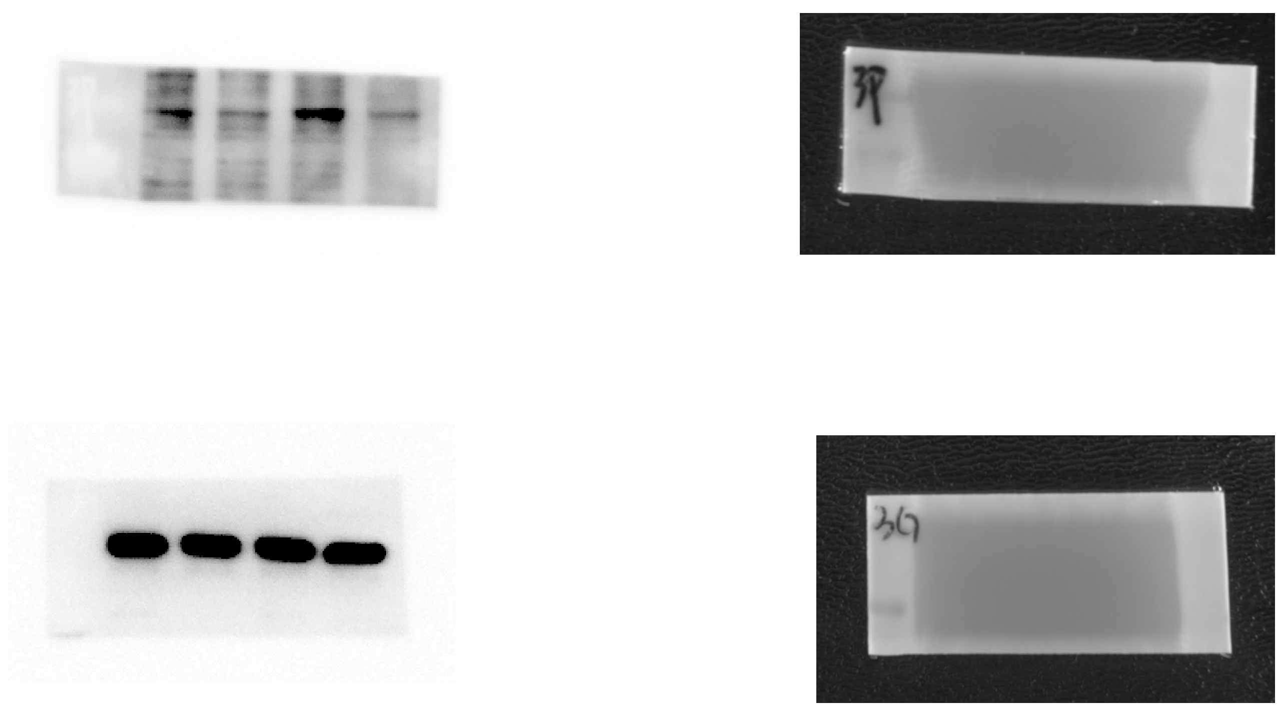

Fig.6 D

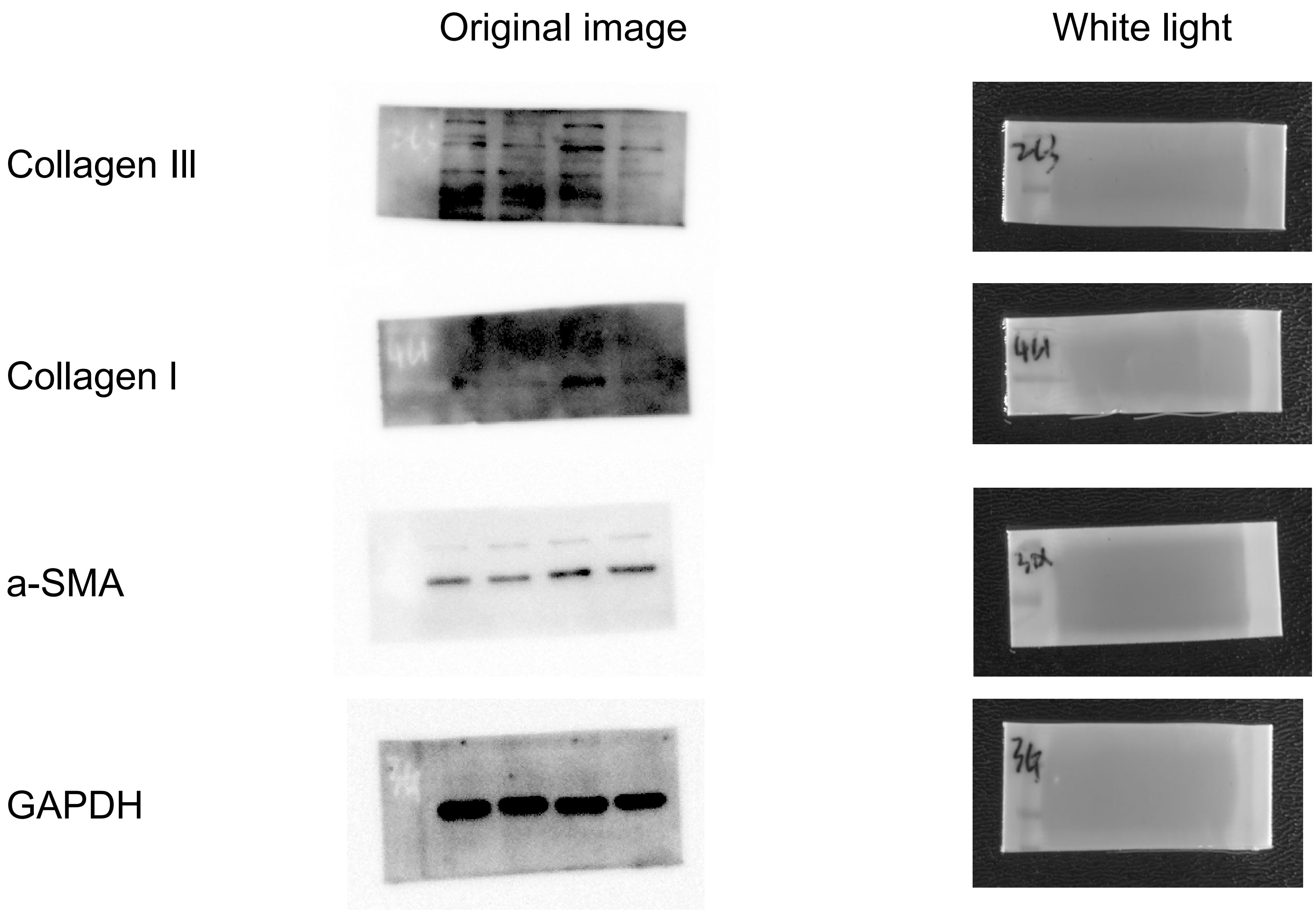
Original image
White light
Collagen III
Collagen I
a-SMA
GAPDH

Fig.6 G

Original image White light

PFKP

GAPDH

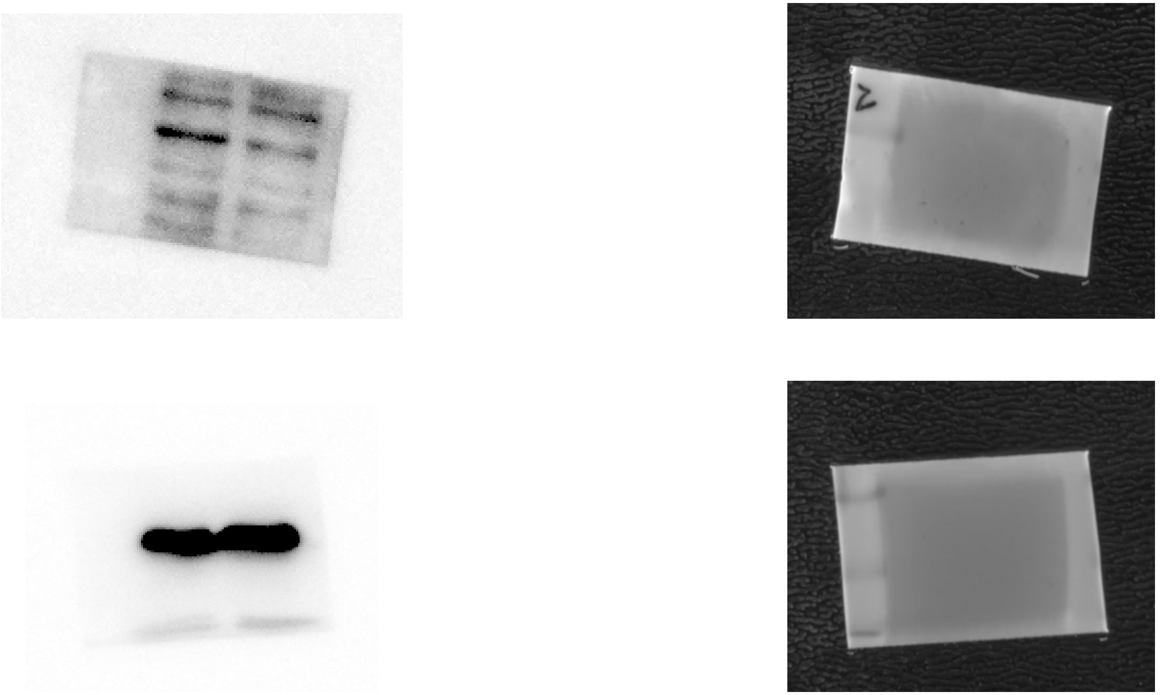

Fig.6 H

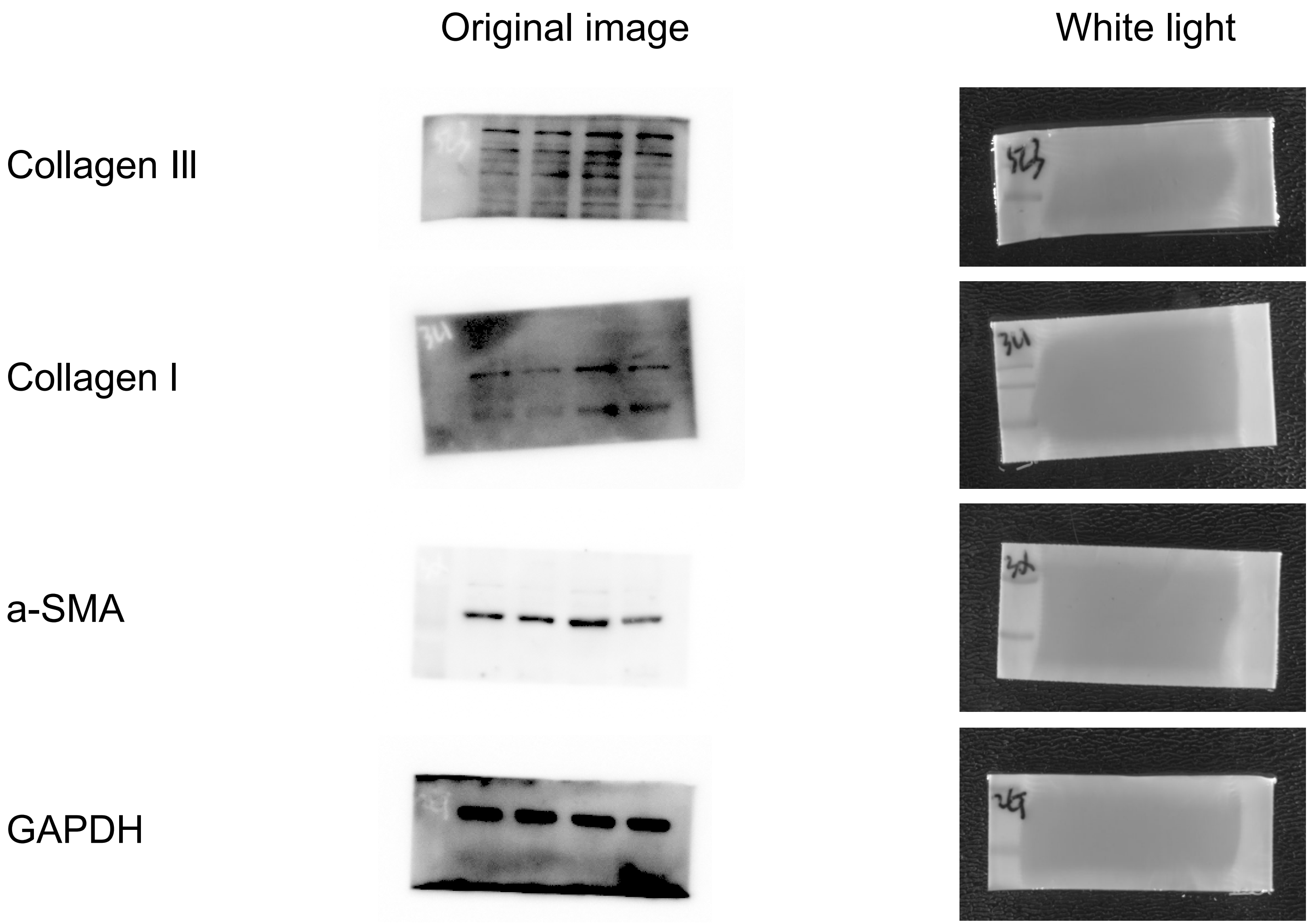
Original image
White light
Collagen III
Collagen I
a-SMA
GAPDH

Supplement Fig.1

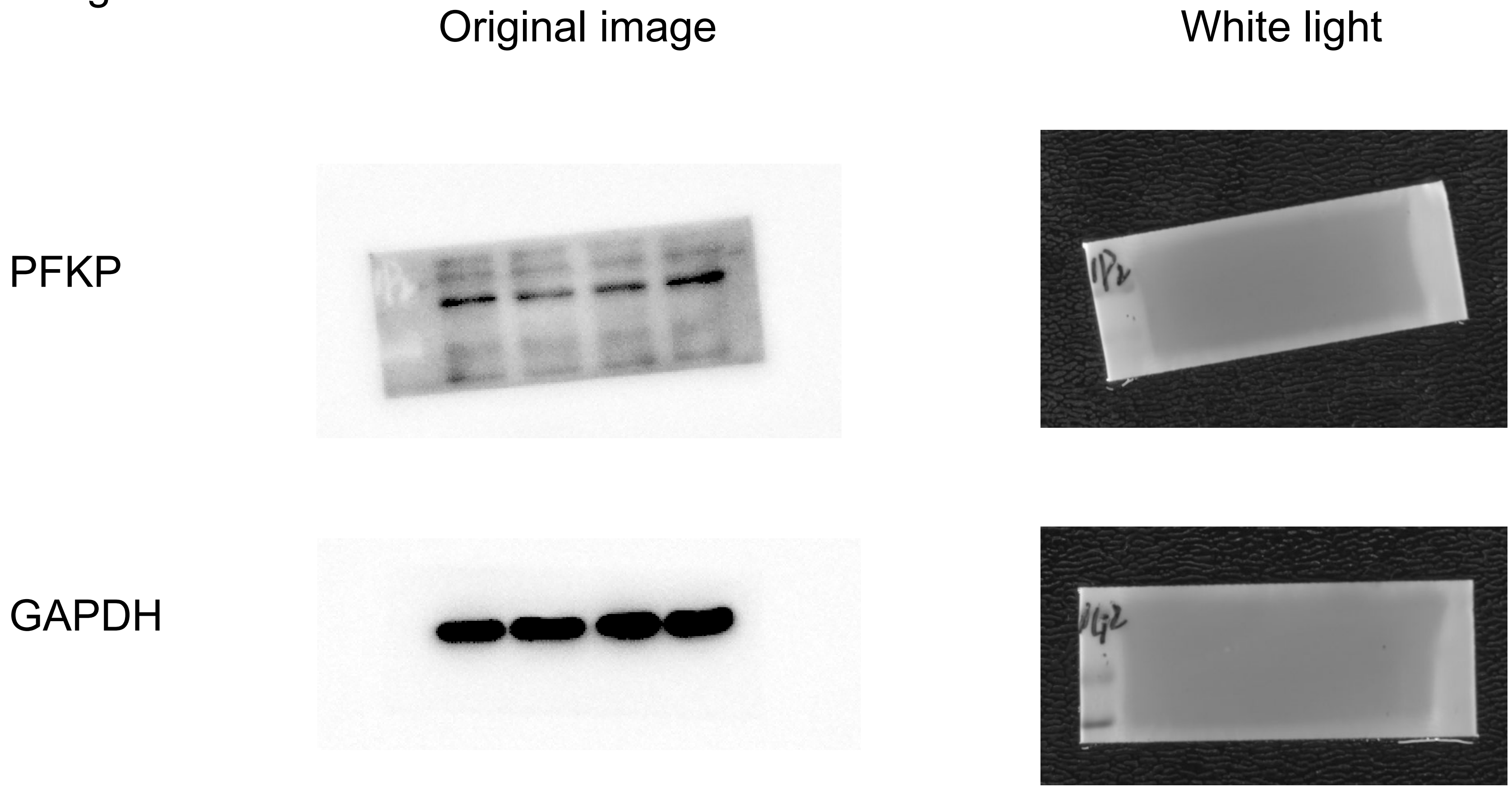

Supplement: Supplementary file 3 [file Supplementaryfile1.pdf]
